# Supplementary material for: Obstacles to detecting isoforms using full-length scRNA-seq data
Source: Genome Biol. 2020 Mar 23;21:74. doi: 10.1186/s13059-020-01981-w (PMC7087381; doi:10.1186/s13059-020-01981-w)
Supplement: Supplementary file 1 — Additional file 1 Supplementary figures and tables. This PDF file contains all of the supplementary figures and tables for this paper. [file 13059_2020_1981_MOESM1_ESM.pdf]

Supplementary Figures and Tables

Jennifer Westoby, Pavel Artemov, Martin Hemberg, Anne Ferguson-Smith

February 8, 2020

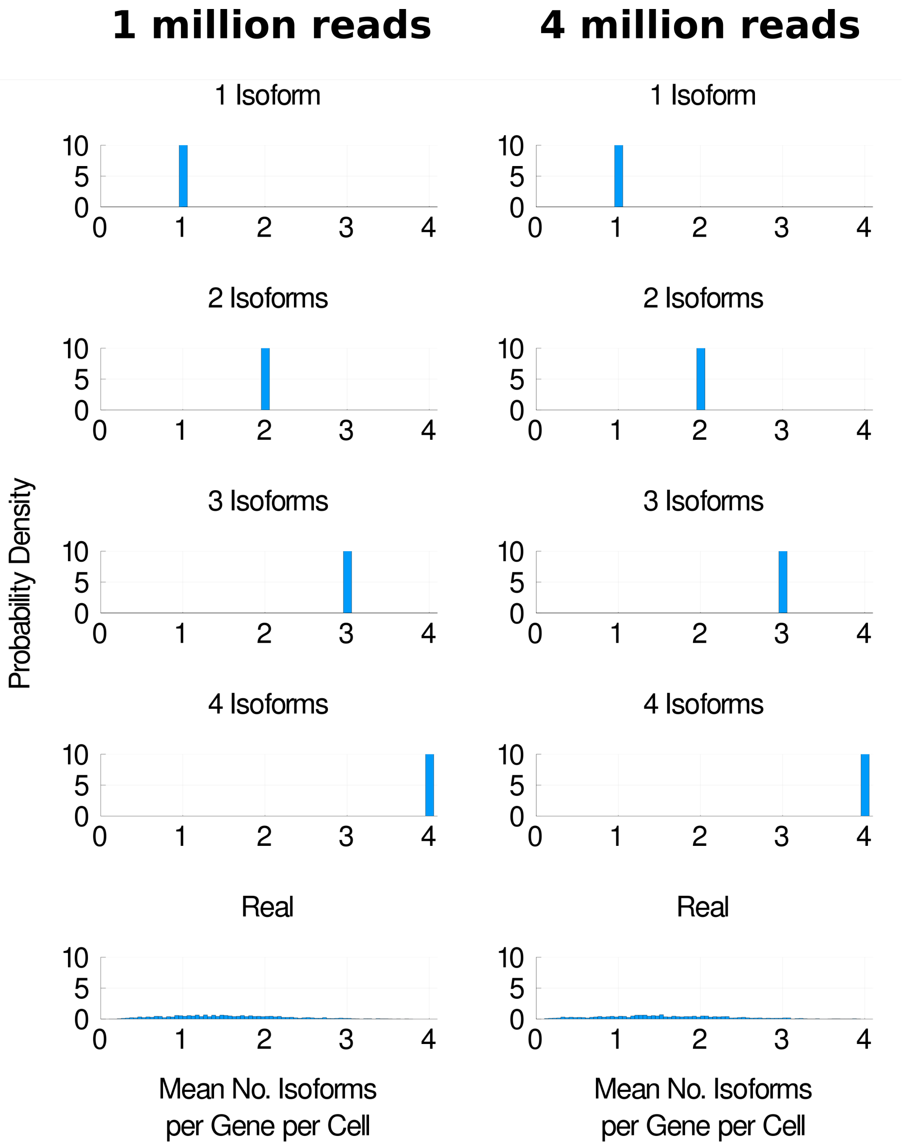

Fig. S1: Negative control model for H1 hESCs. In the simulation results displayed, no dropouts or quantification errors were simulated. The simulation procedure was otherwise unchanged.

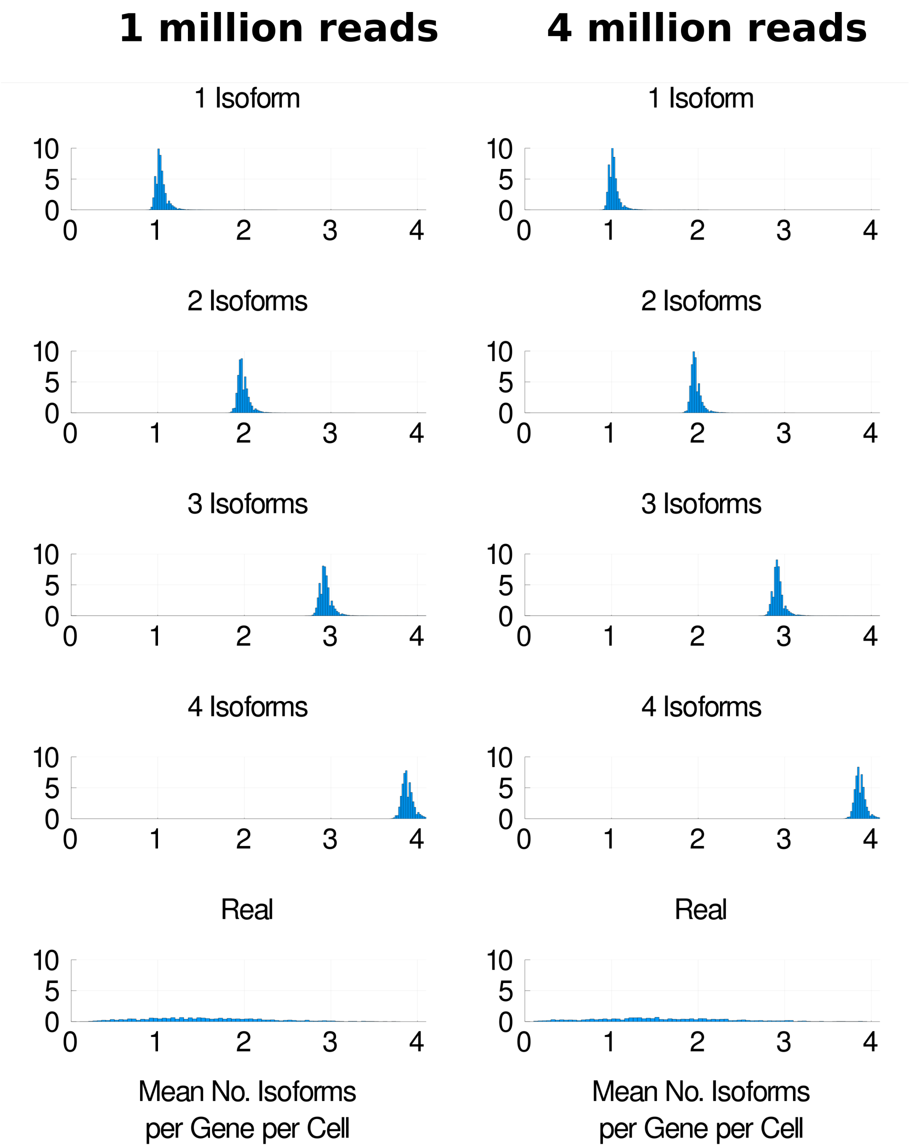

Fig. S2: Negative control model for H1 hESCs. In the simulation results displayed, no dropouts were simulated. The simulation procedure was otherwise unchanged.

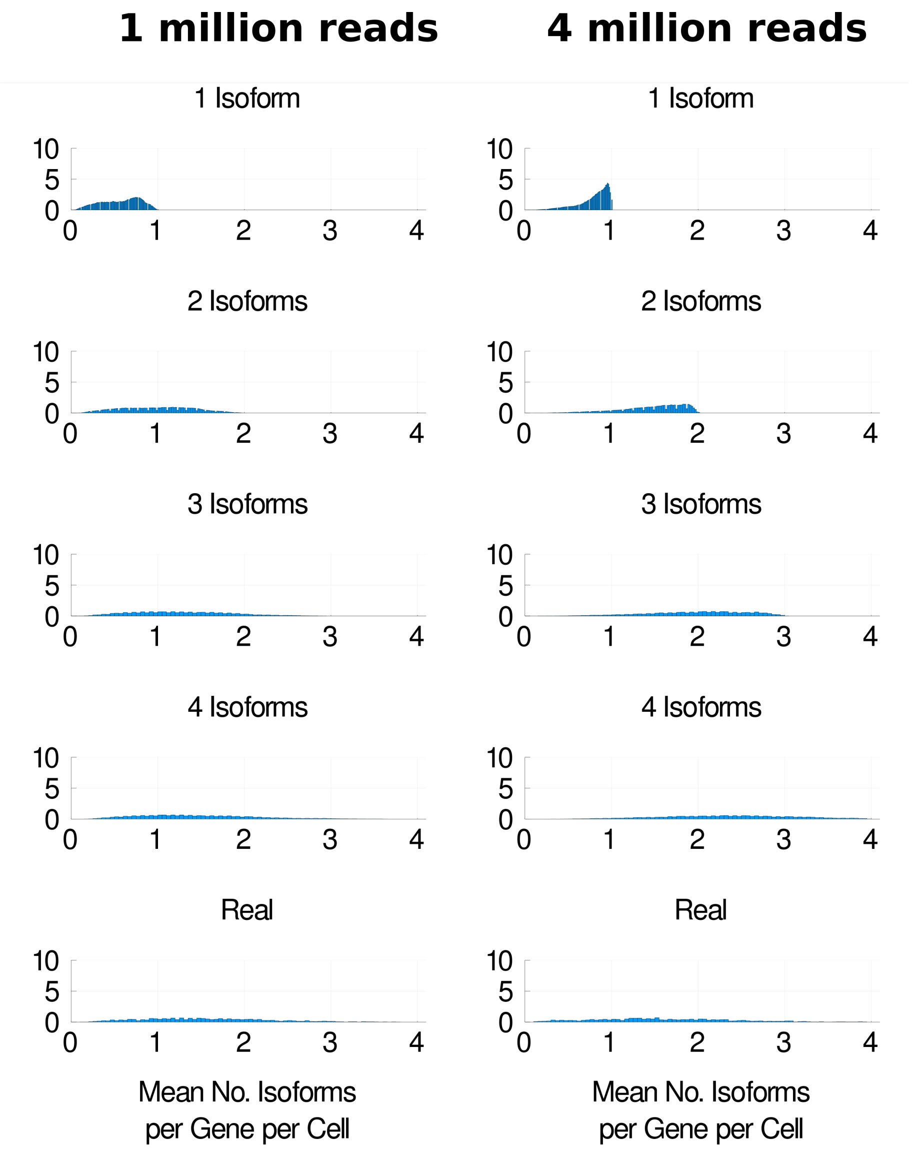

Fig. S3: Negative control model for H1 hESCs. In the simulation results displayed, no quantification errors were simulated. The simulation procedure was otherwise unchanged.

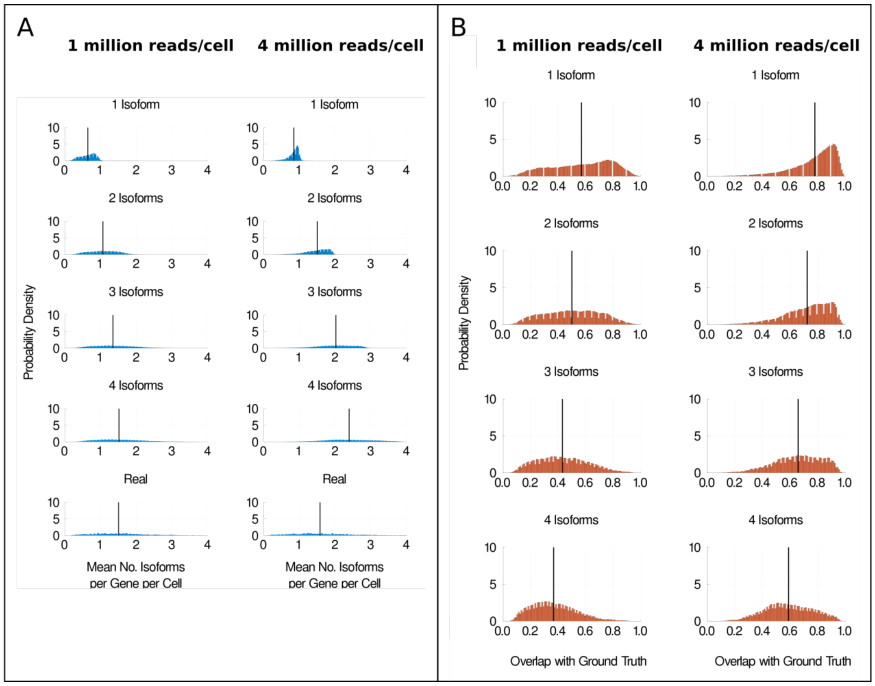

Fig. S4: The effect of sequencing depth on isoform detection. **a** Distributions of the mean number of isoforms detected per gene per cell for H9 hESCs whose cDNA was split and sequenced at approximately 1 million reads per cell or 4 million reads per cell on average. **b** Distributions of the overlap fraction with the ground truth.

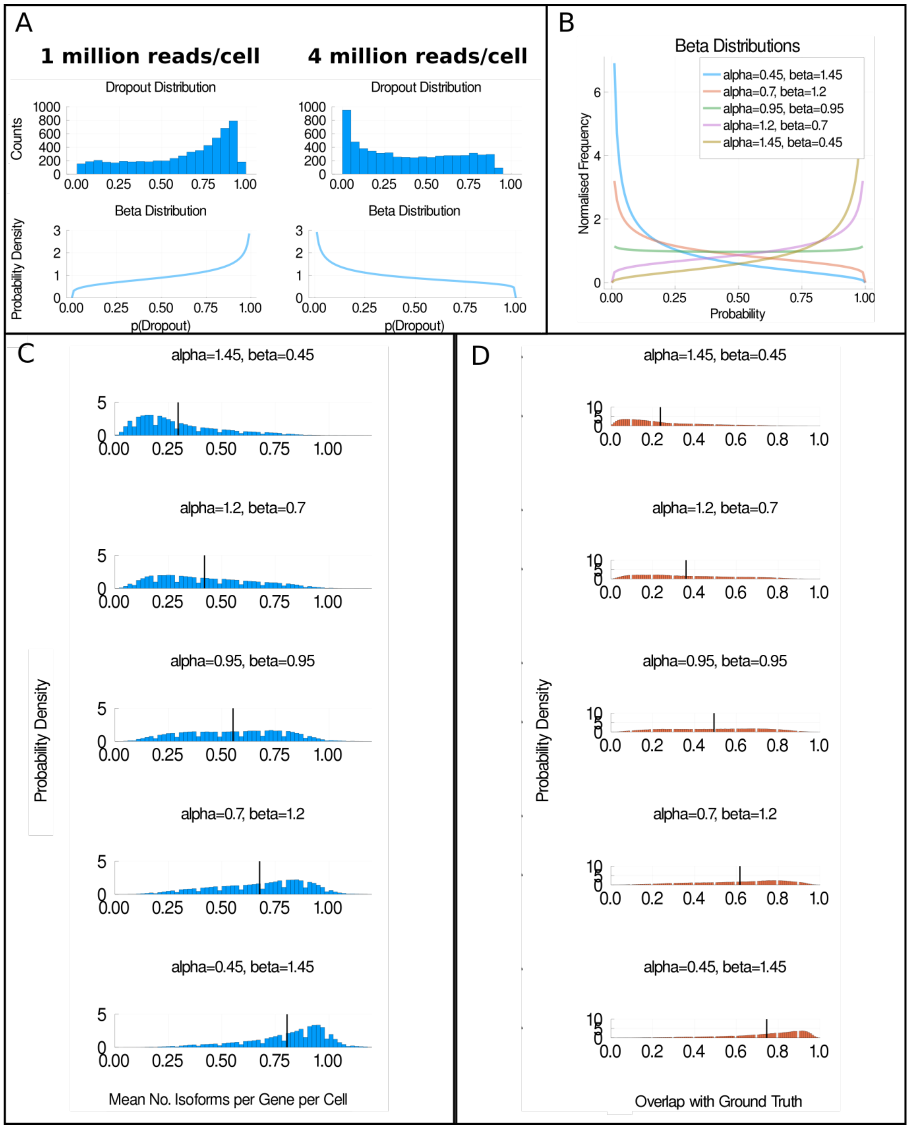

Fig. S5: The impact of dropouts on isoform detection. **a** shows the distribution of the probabilities of dropouts (p(Dropout)) in each group of H9 hESCs and an approximation of these distributions using a Beta distribution. At 1 million reads per cell, $\alpha = 1.31$ and $\beta = 0.74$ in the approximated Beta distribution. At 4 million reads per cell, $\alpha = 0.72$ and $\beta = 1.03$ in the approximated Beta distribution. **b** shows five Beta Distributions from which dropout probabilities were sampled from in the simulations used to generate **c** and **d**. In **c**, the distribution of the mean number of isoforms detected per gene per cell is shown for simulations in which one isoform was produced per gene per cell. Each plot corresponds to a simulation in which dropout probabilities were sampled from one of the distributions shown in **b**. **d** shows the overlap fraction with the ground truth for each simulation. Plots shown in **c** & **d** are for H9 hESCs sequenced at 4 million reads per cell.

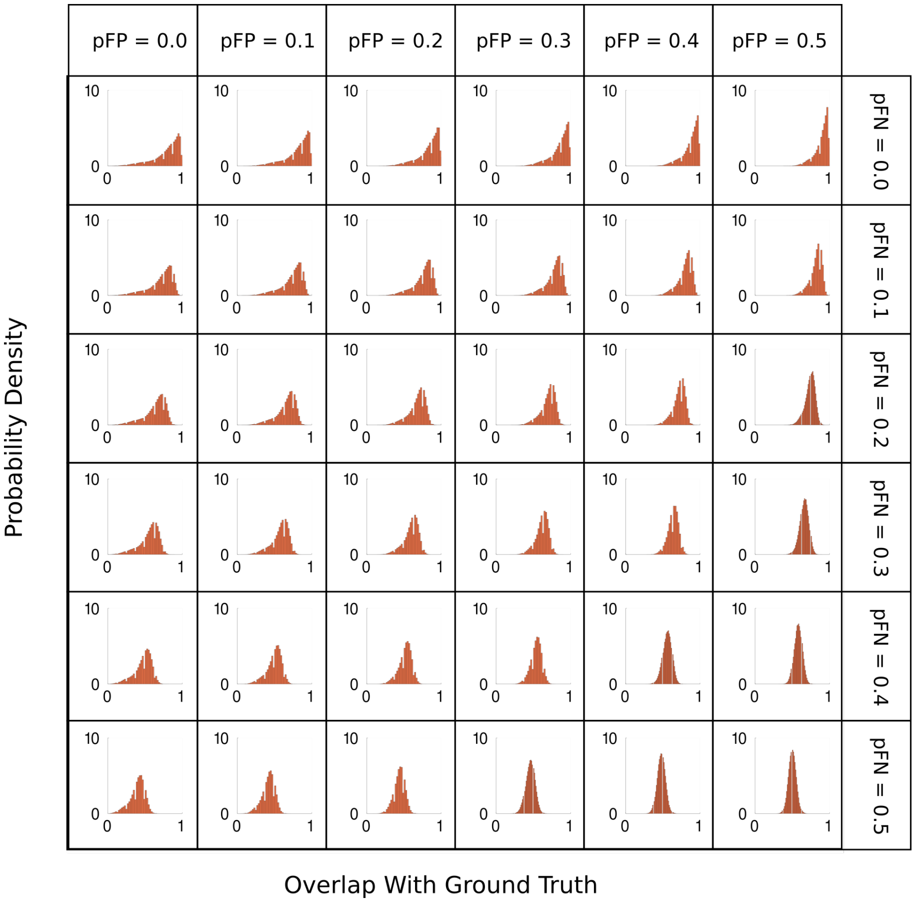

Fig. S6: The impact of quantification errors on isoform detection. Distributions of the overlap fraction with the ground truth when one isoform is expressed per gene per cell. The probability of false positives (pFP) increases from left to right and the probability of false negatives (pFN) increases from top to bottom. The dataset shown is H1 hESCs whose cDNA was split and sequenced at approximately 4 million reads per cell on average.

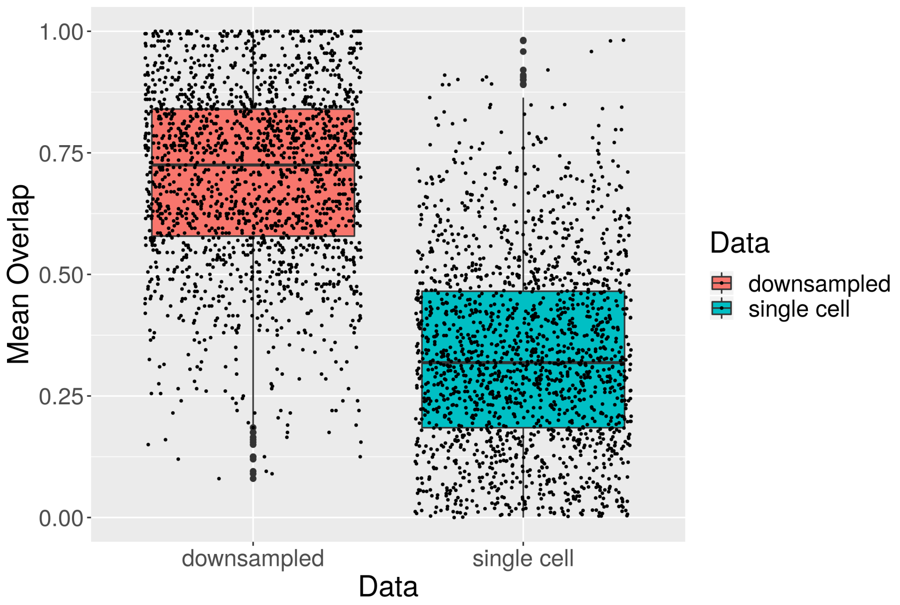

Fig. S7: Boxplots of the mean overlap for each gene in the downsampled bulk and matched scRNA-seq datasets. The mean overlaps for each gene are overlaid on the boxplots as black points. Plots shown for Kolodziejczyk et al. mESCs cultured in standard 2i media + LIF [1].

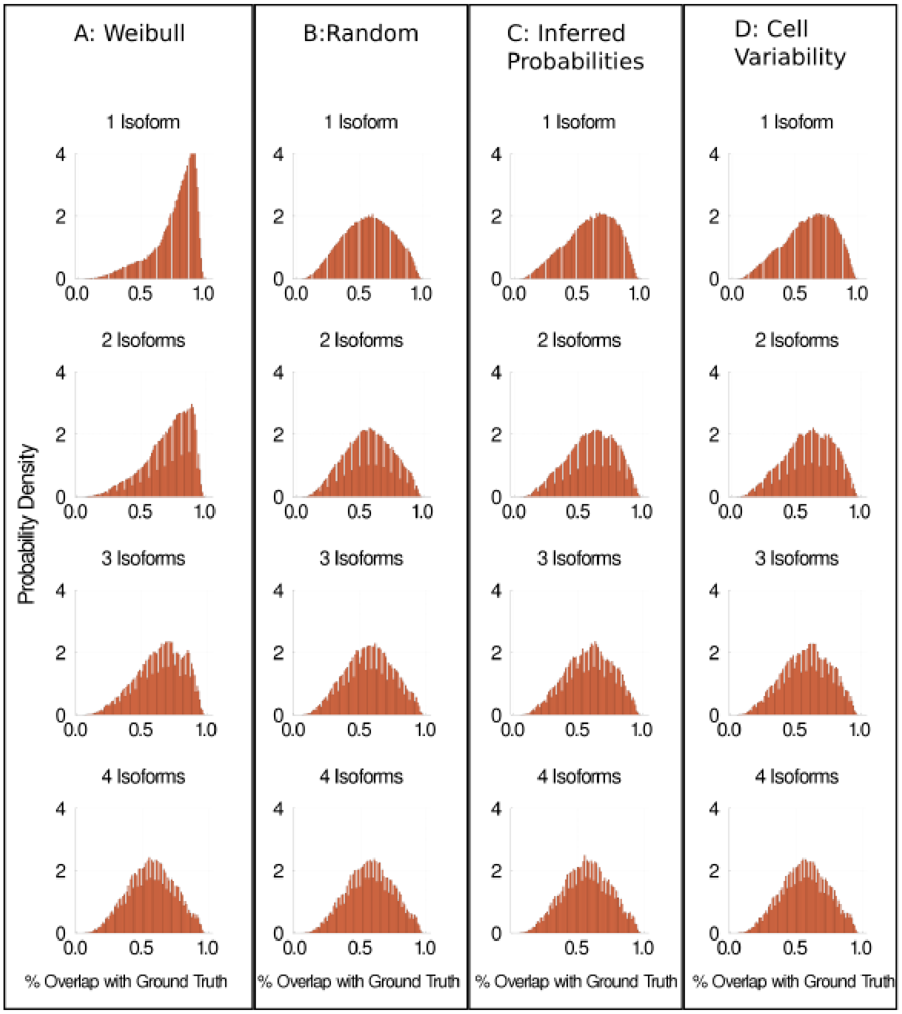

Fig. S8: Distributions of the overlap fraction with the ground truth when the **a** Weibull model [2, 3], **b** random model, **c** inferred probabilities model and **d** cell variability model of isoform choice is used. All distributions are for H1 cells sequenced at approximately 4 million reads per cell. See the main text for a detailed description of each model.

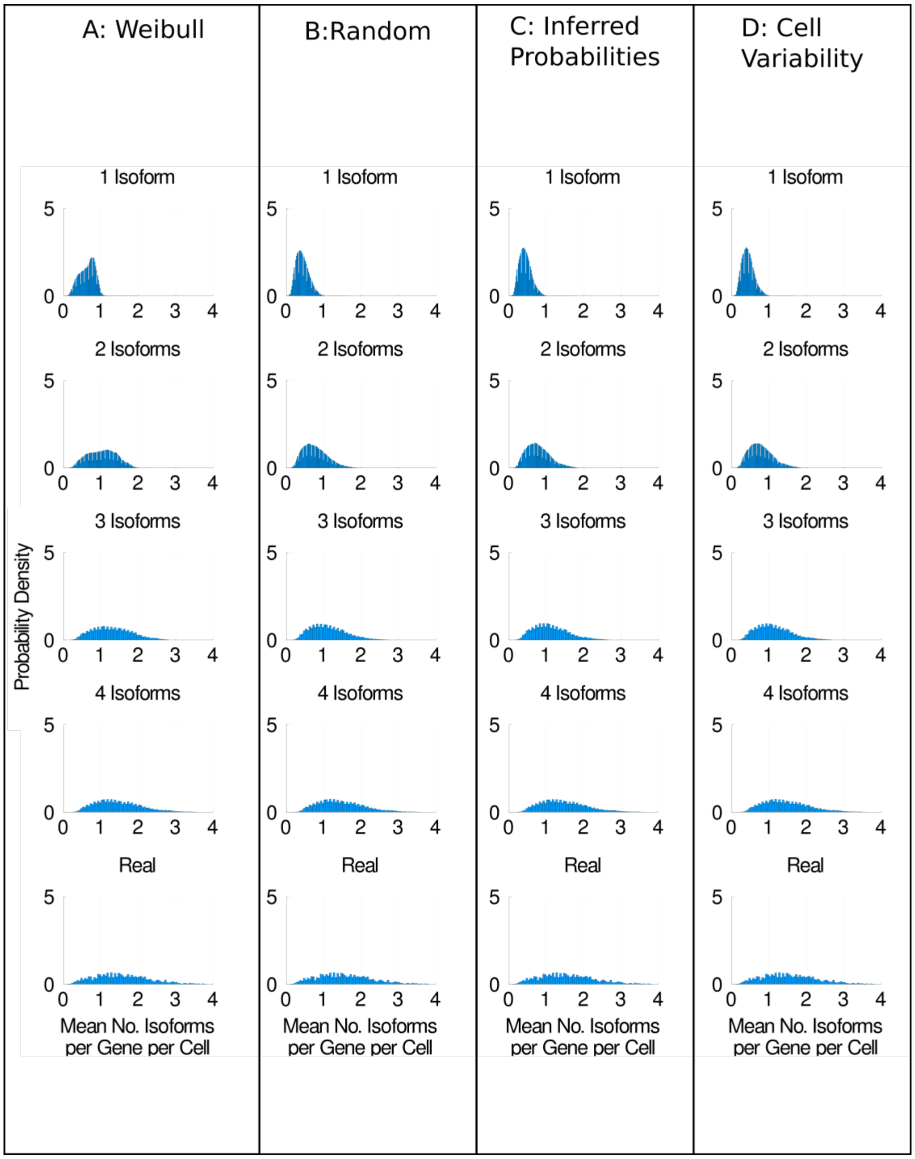

Fig. S9: Different models of isoform choice alter our ability to detect isoforms. **a** Distributions of the mean number of isoforms detected per gene per cell for H1 hESCs sequenced at approximately 1 million reads per cell using the Weibull model of isoform choice [2, 3]. **b** shows the same distributions when the random model is used. **c** shows the distributions when the inferred probabilities model is used. **d** shows the distributions when the cell variability model is used. See the main text for a detailed description of each model.

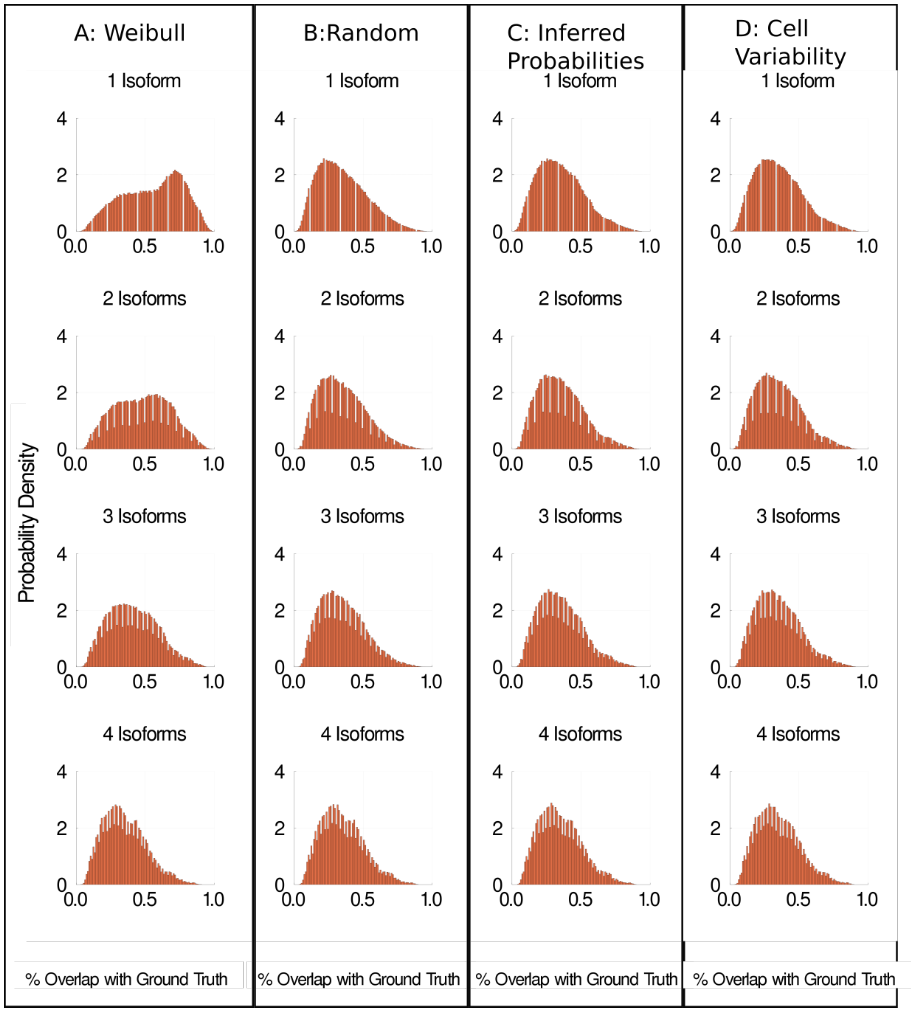

Fig. S10: Different models of isoform choice alter our ability to detect isoforms. **a** Distributions of overlap fraction with the ground truth for H1 hESCs sequenced at approximately 1 million reads per cell using the Weibull model of isoform choice [2, 3]. **b** shows the same distributions when the random model is used. **c** shows the distributions when the inferred probabilities model is used. **d** shows the distributions when the cell variability model is used. See the main text for a detailed description of each model.

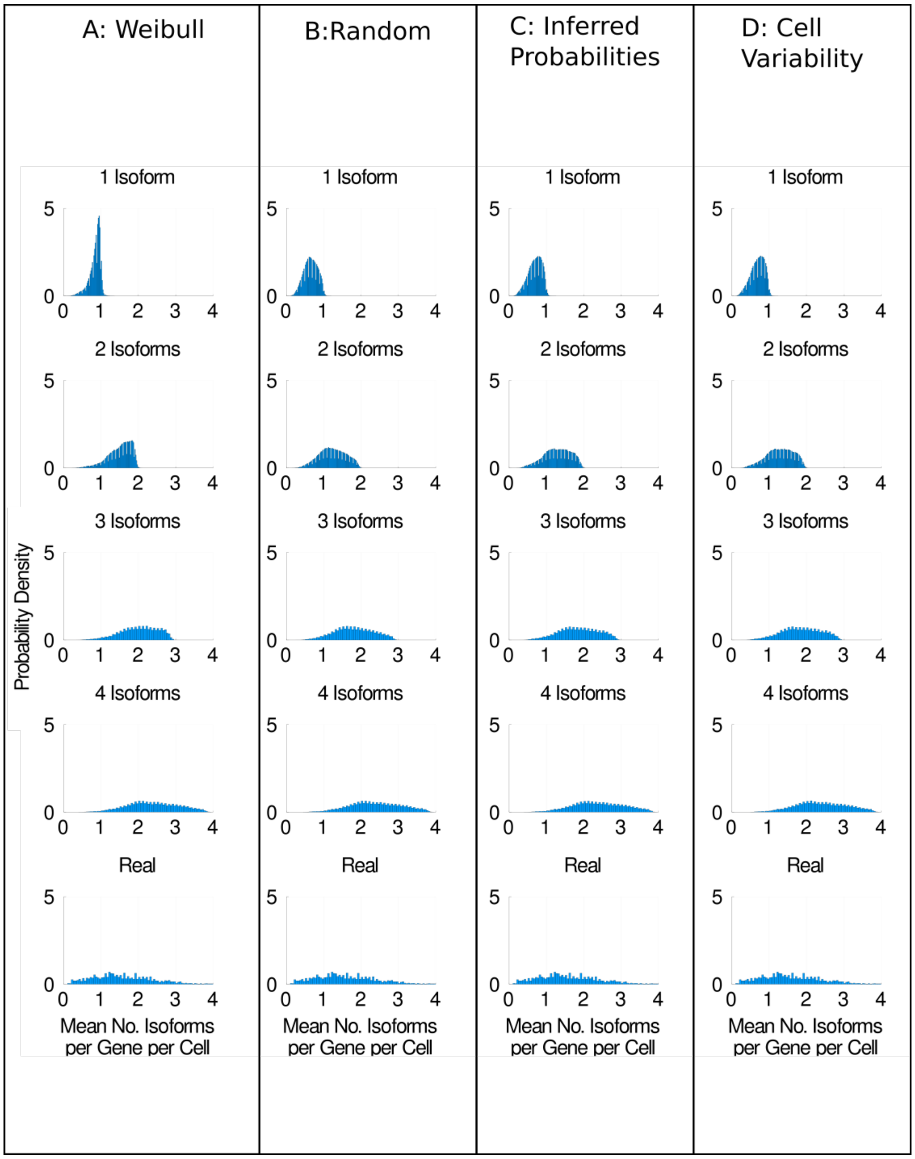

Fig. S11: Different models of isoform choice alter our ability to detect isoforms. **a** Distributions of the mean number of isoforms detected per gene per cell for H9 hESCs sequenced at approximately 4 million reads per cell using the Weibull model of isoform choice [2, 3]. **b** shows the same distributions when the random model is used. **c** shows the distributions when the inferred probabilities model is used. **d** shows the distributions when the cell variability model is used. See the main text for a detailed description of each model.

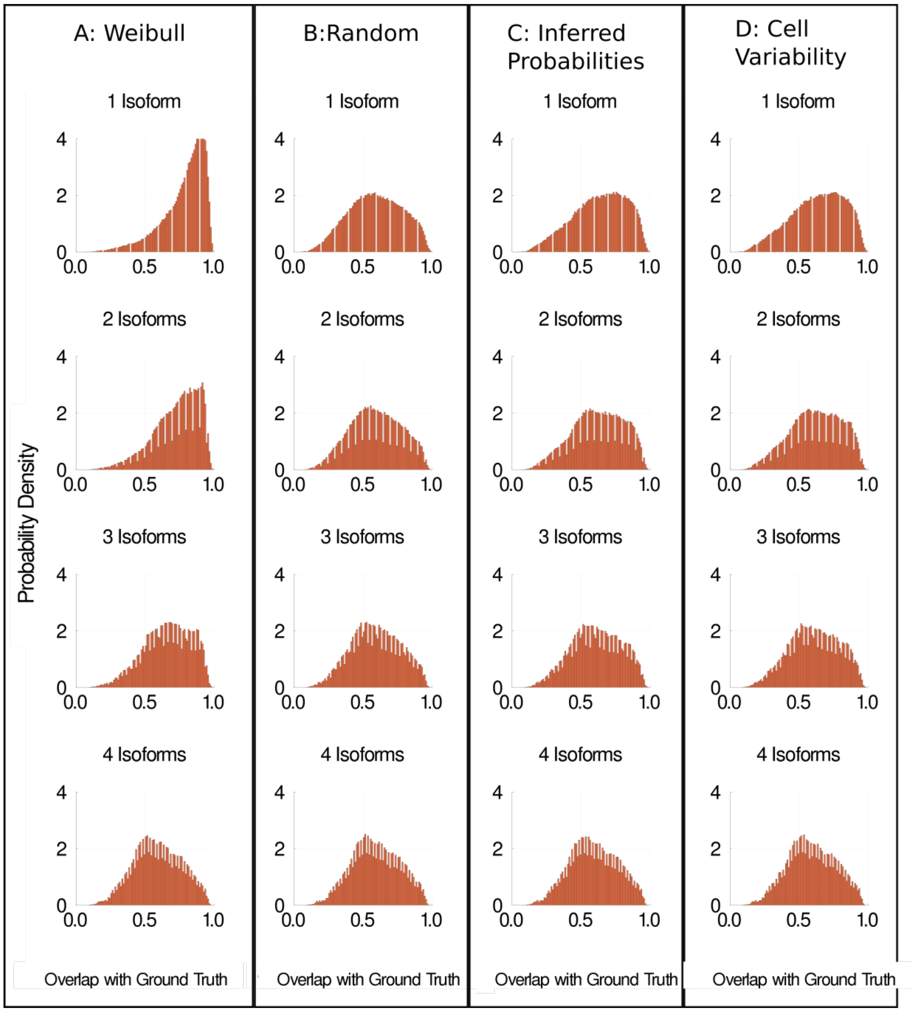

Fig. S12: Different models of isoform choice alter our ability to detect isoforms. **a** Distributions of overlap fraction with the ground truth for H9 hESCs sequenced at approximately 4 million reads per cell using the Weibull model of isoform choice [2, 3]. **b** shows the same distributions when the random model is used. **c** shows the distributions when the inferred probabilities model is used. **d** shows the distributions when the cell variability model is used. See the main text for a detailed description of each model.

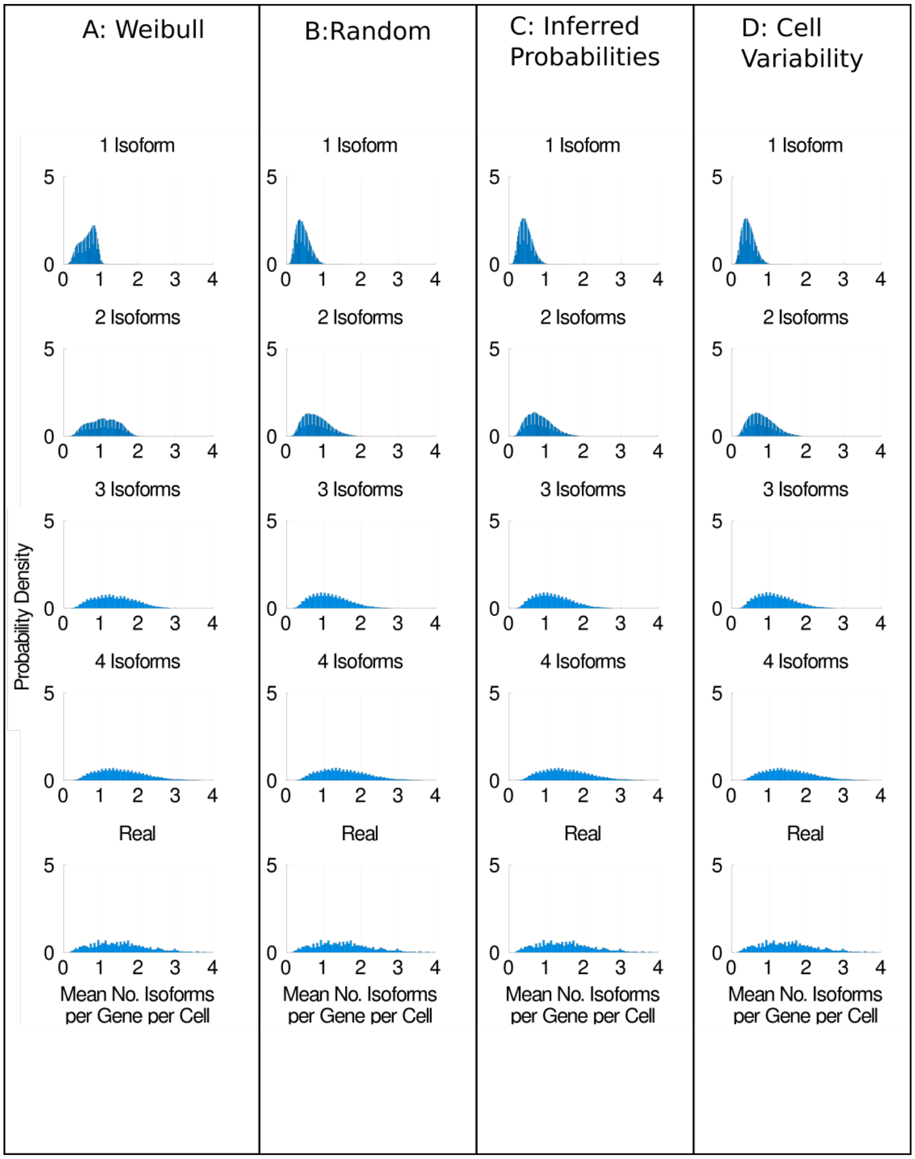

Fig. S13: Different models of isoform choice alter our ability to detect isoforms. **a** Distributions of the mean number of isoforms detected per gene per cell for H9 hESCs sequenced at approximately 1 million reads per cell using the Weibull model of isoform choice [2, 3]. **b** shows the same distributions when the random model is used. **c** shows the distributions when the inferred probabilities model is used. **d** shows the distributions when the cell variability model is used. See the main text for a detailed description of each model.

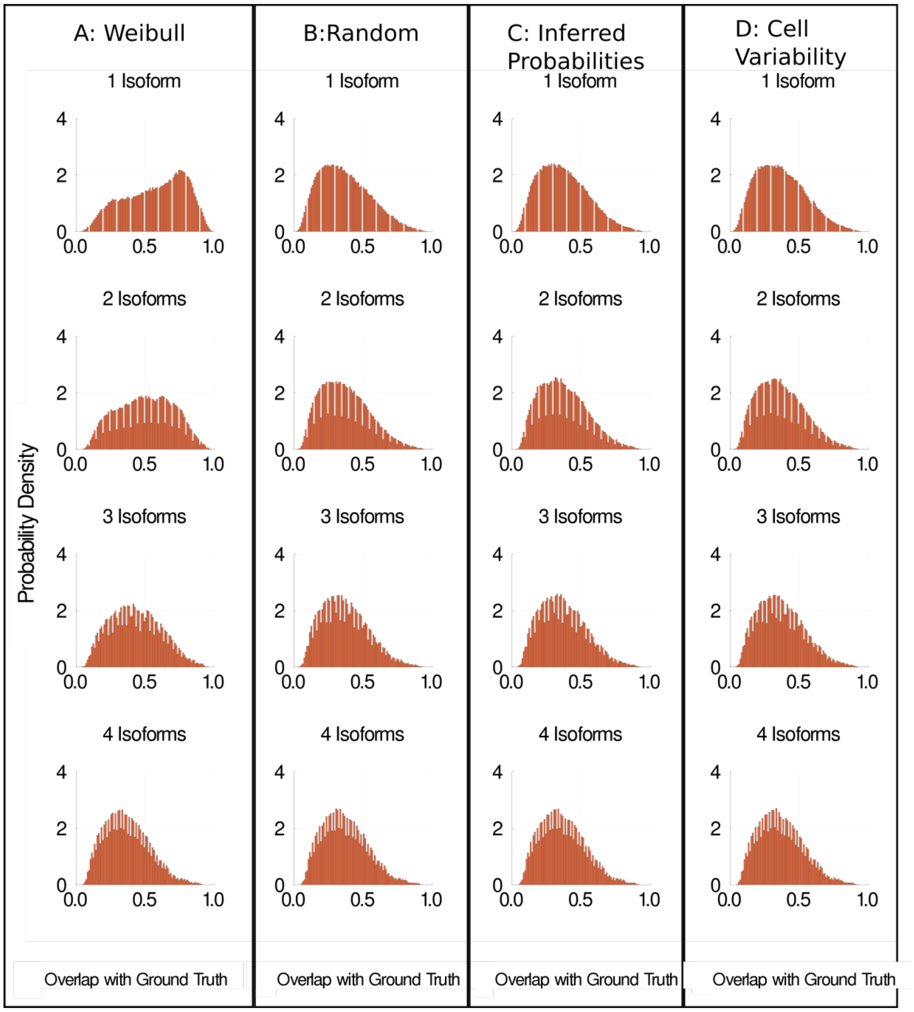

Fig. S14: Different models of isoform choice alter our ability to detect isoforms. **a** Distributions of overlap fraction with the ground truth for H9 hESCs sequenced at approximately 1 million reads per cell using the Weibull model of isoform choice [2, 3]. **b** shows the same distributions when the random model is used. **c** shows the distributions when the inferred probabilities model is used. **d** shows the distributions when the cell variability model is used. See the main text for a detailed description of each model.

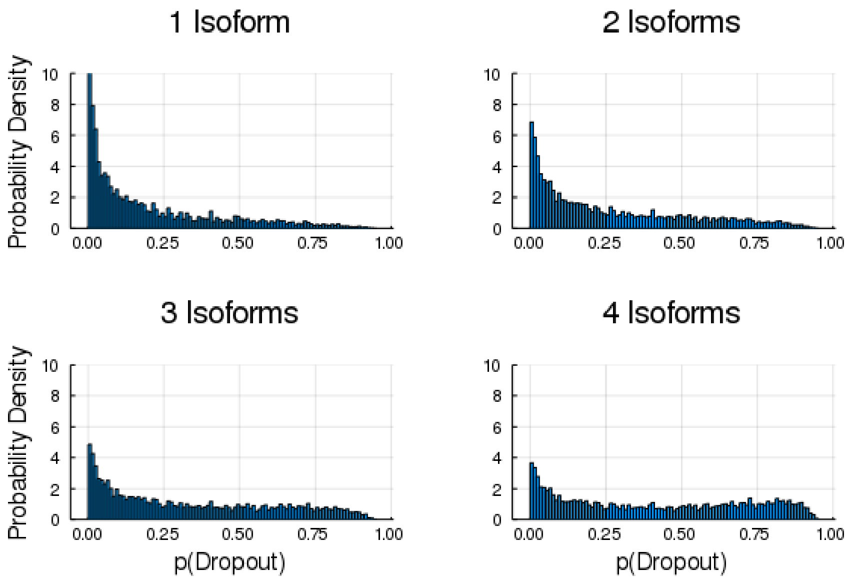

Fig. S15: Distributions of the probabilities of dropouts for the isoforms selected by the Weibull model when one, two, three and four isoforms were picked by the model.

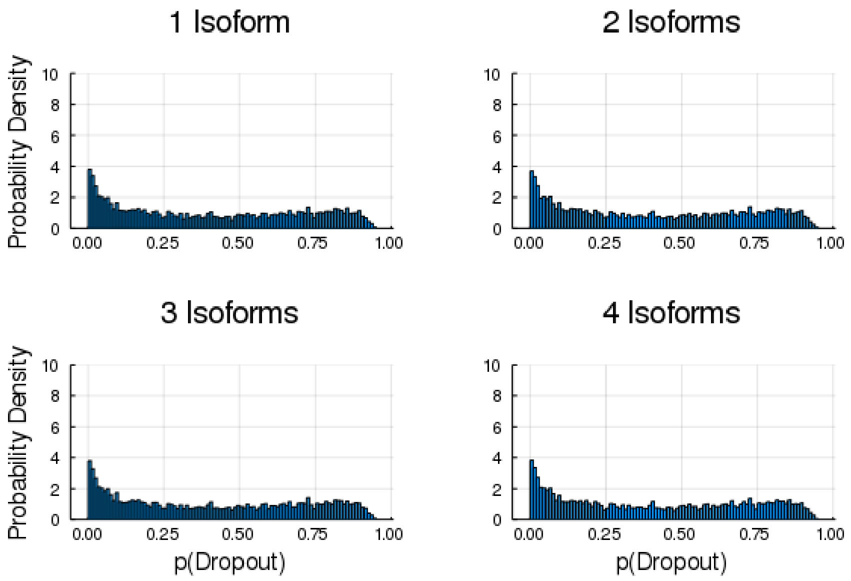

Fig. S16: Distributions of the probabilities of dropouts for the isoforms selected by the Random model when one, two, three and four isoforms were picked by the model.

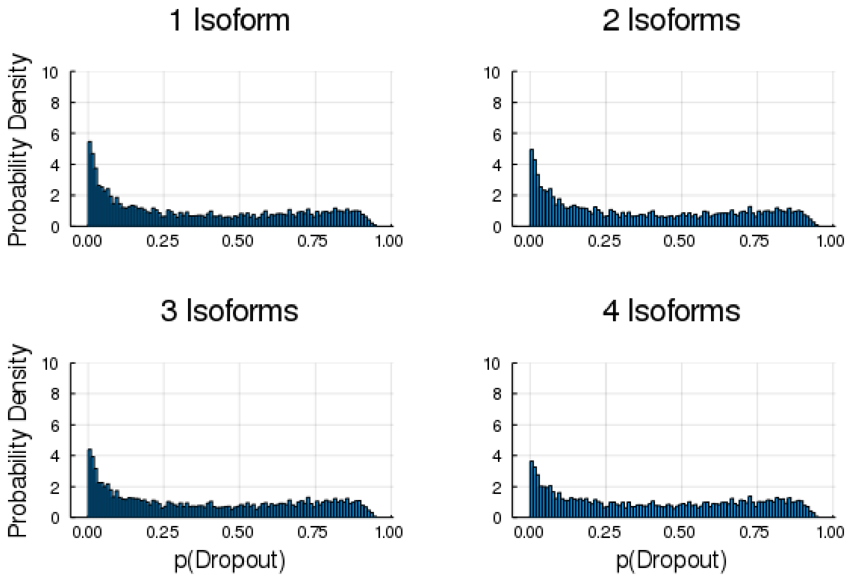

Fig. S17: Distributions of the probabilities of dropouts for the isoforms selected by the inferred probabilities model when one, two, three and four isoforms were picked by the model.

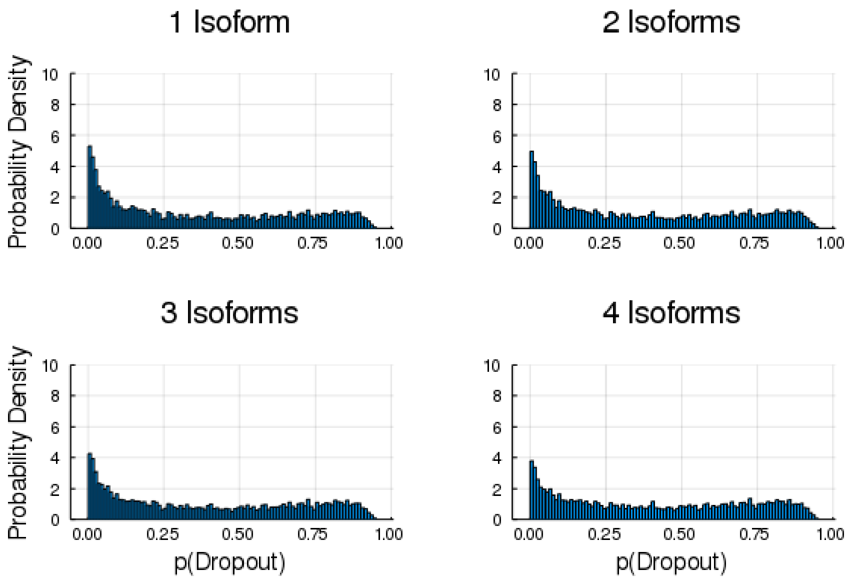

Fig. S18: Distributions of the probabilities of dropouts for the isoforms selected by the cell variable model when one, two, three and four isoforms were picked by the model.

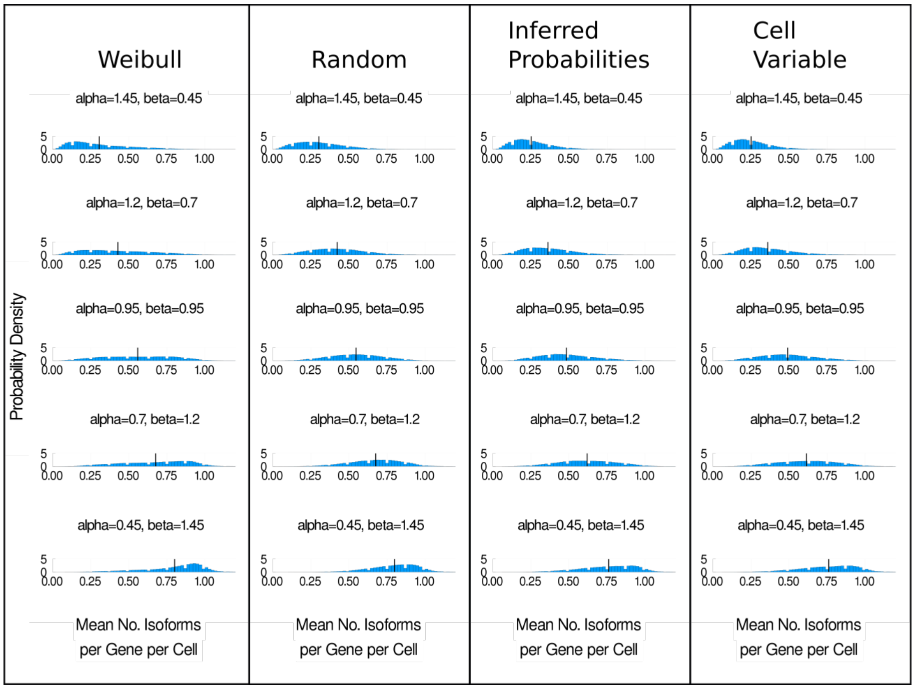

Fig. S19: Distributions of the mean number of isoforms detected per gene per cell under different isoform choice models when dropout probabilities are sampled from the Beta distributions in Figure 3B in the main text.

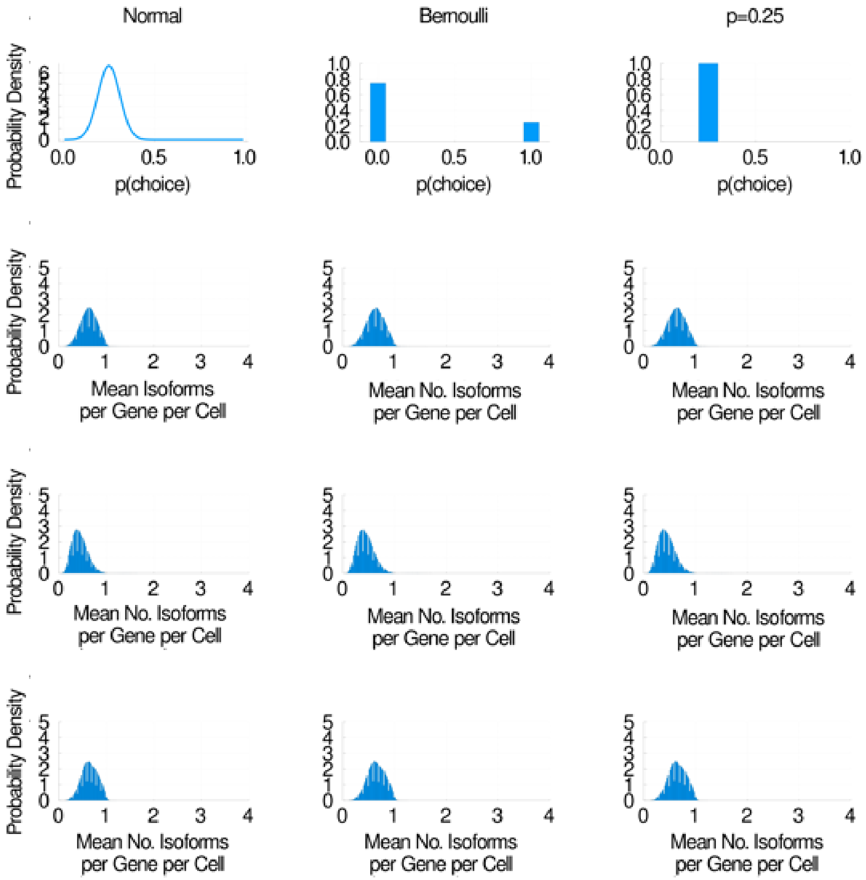

Fig. S20: Some models of isoform choice are more plausible than others. We model the probability of picking any given isoform as a Normal distribution, a Bernoulli distribution and a constant probability, all with the same mean (0.25) (top row of graphs). In the following rows, we show the distributions of the mean number of isoforms per gene per cell detected when each model of isoform choice is used. The second row is H1 hESCs sequenced at 4 million reads, the third row is H9 hESCs sequenced at 1 million reads, the fourth row is H9 hESCs sequenced at 4 million reads.

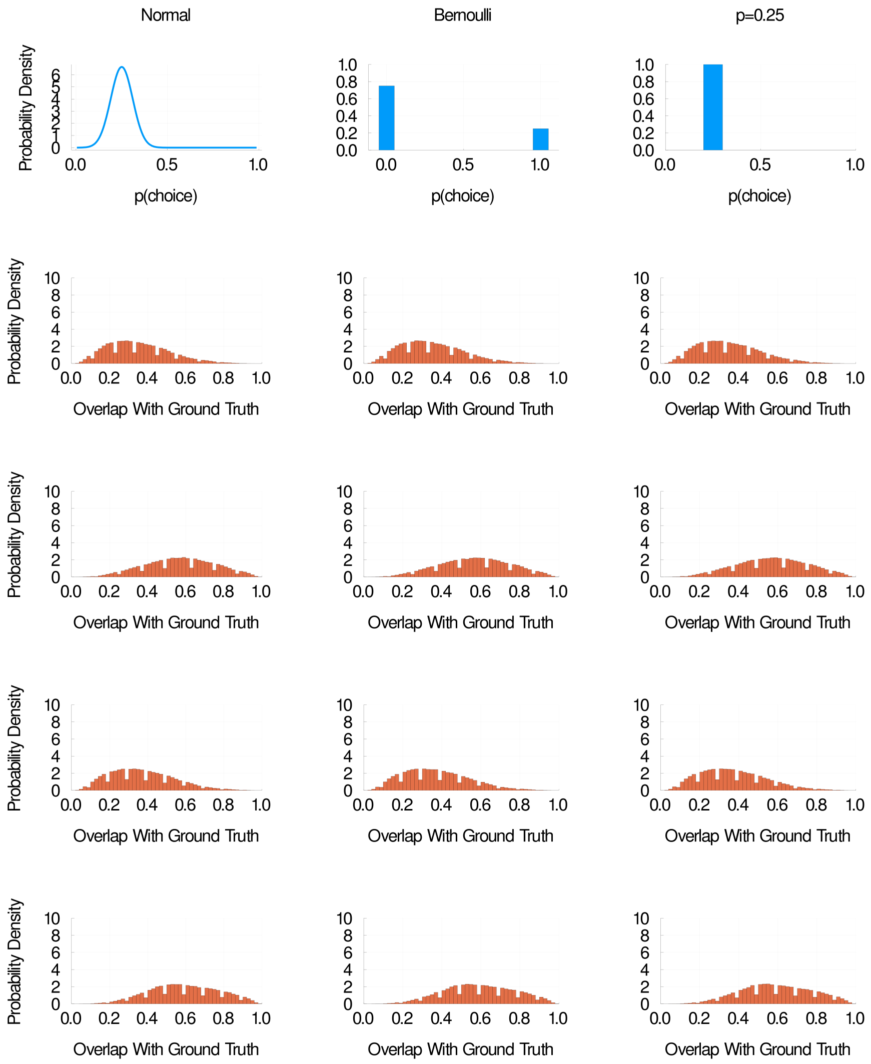

Fig. S21: Some models of isoform choice are more plausible than others. We model the probability of picking any given isoform as a Normal distribution, a Bernoulli distribution and a constant probability, all with the same mean (0.25) (top row of graphs). In the following rows, we show the distributions of the overlap fraction when each model of isoform choice is used. The second row is H1 hESCs sequenced at 1 million reads per cell, the third row is H1 hESCs sequenced at 4 million reads, the fourth row is H9 hESCs sequenced at 1 million reads, the fifth row is H9 hESCs sequenced at 4 million reads.

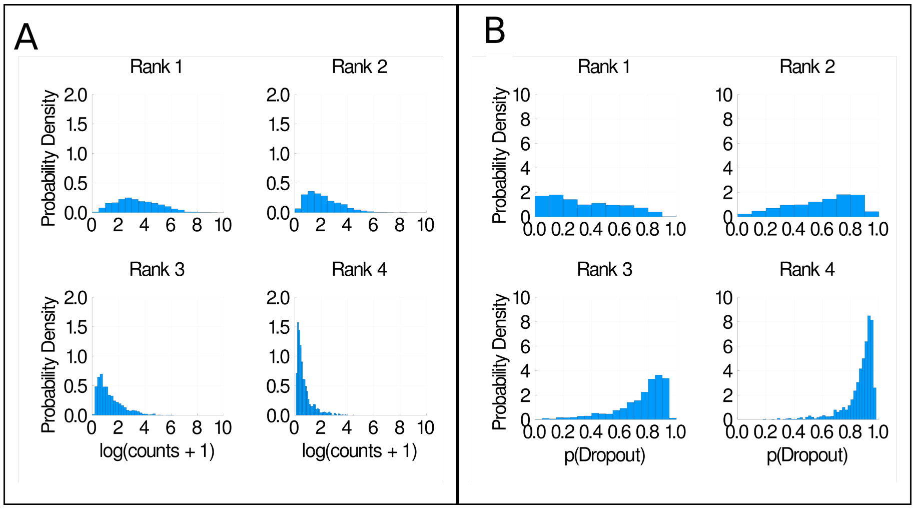

Fig. S22: **a** Histograms of mean isoform expression, ordered by isoform rank. **b** Histograms of dropout probability, ordered by isoform rank. All plots shown are for H1 hESCs sequenced at 4 million reads per cell.

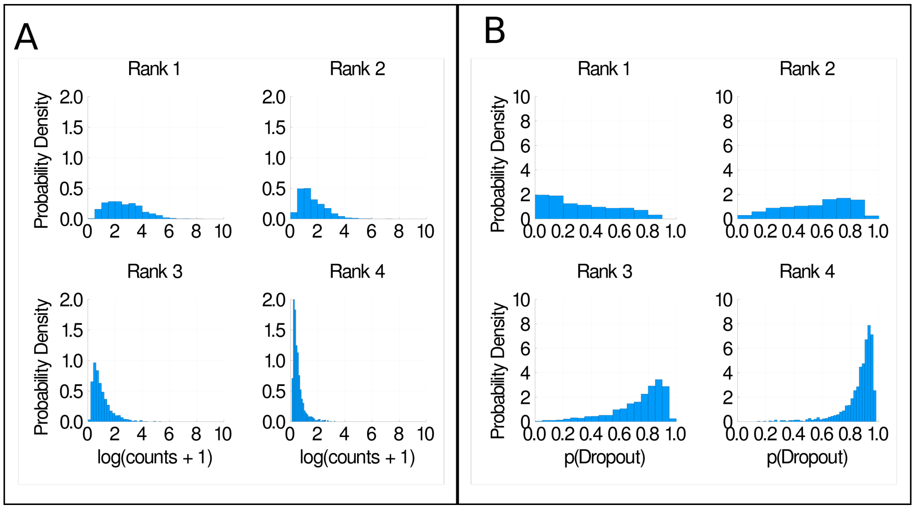

Fig. S23: **a** Histograms of mean isoform expression, ordered by isoform rank. **b** Histograms of dropout probability, ordered by isoform rank. All plots shown are for H9 hESCs sequenced at 1 million reads per cell.

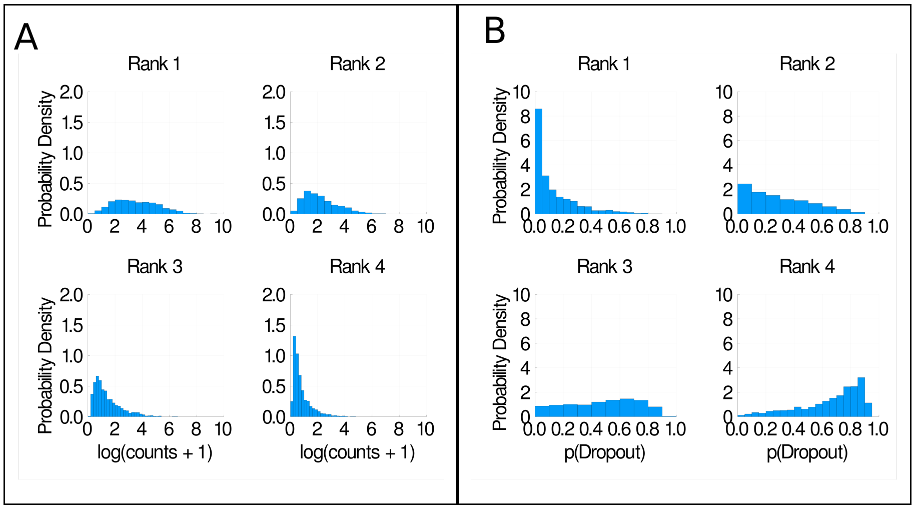

Fig. S24: **a** Histograms of mean isoform expression, ordered by isoform rank. **b** Histograms of dropout probability, ordered by isoform rank. All plots shown are for H9 hESCs sequenced at 4 million reads per cell.

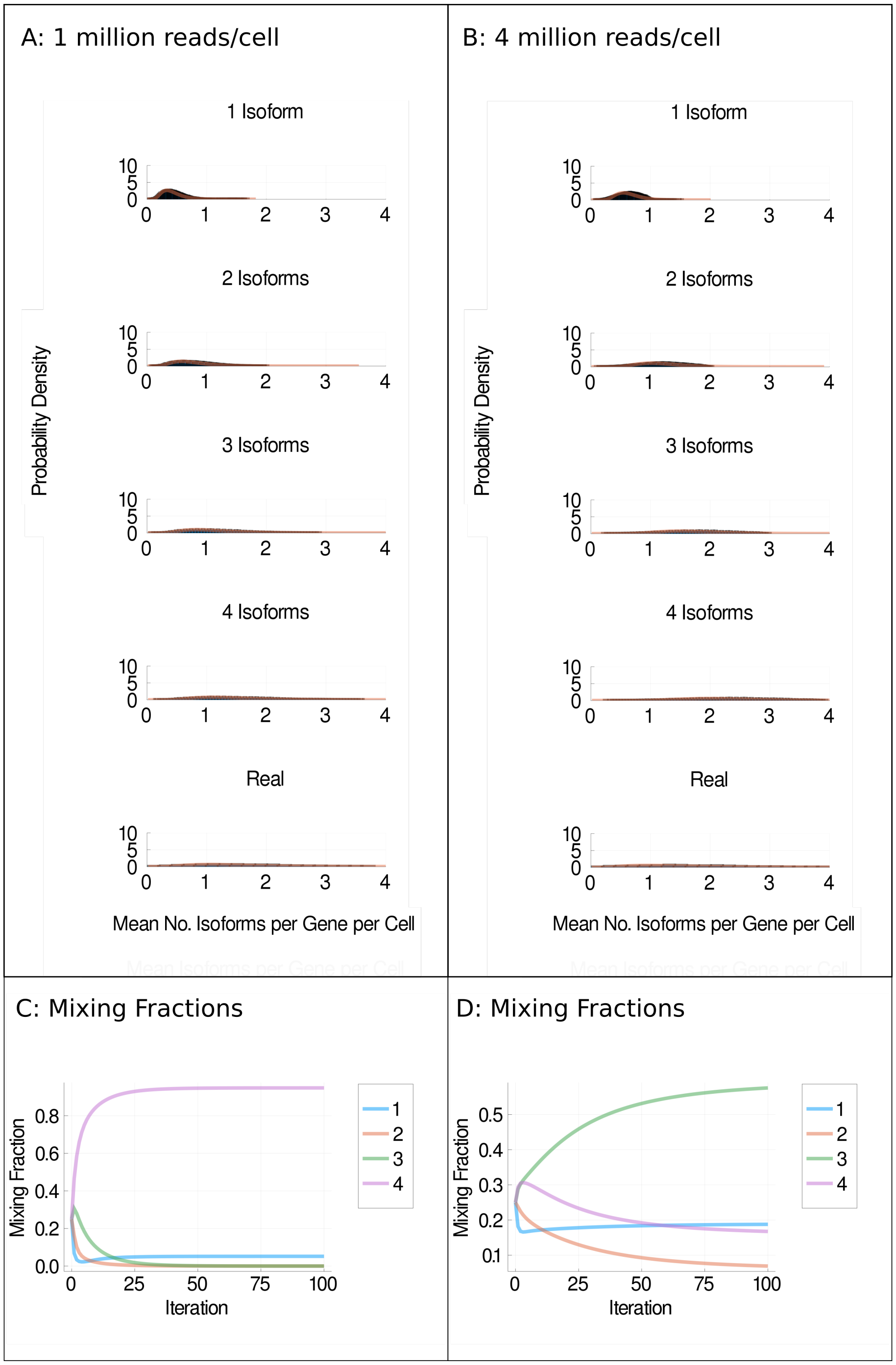

Fig. S25: Mixture models. **a** and **b** Distributions of detected isoforms per gene per cell (blue) and log normal fitted distributions (orange) for H1 cells sequenced at 1 million reads per cell (**a**) or 4 million reads per cell (**b**) under the random model [2]. **c** and **d** Mixing fractions vs iterations of expectation maximisation for 1 million reads per cell (**c**) and 4 million reads per cell (**d**). Each coloured line represents the distributions for one, two, three or four isoforms being simulated as expressed per gene per cell.

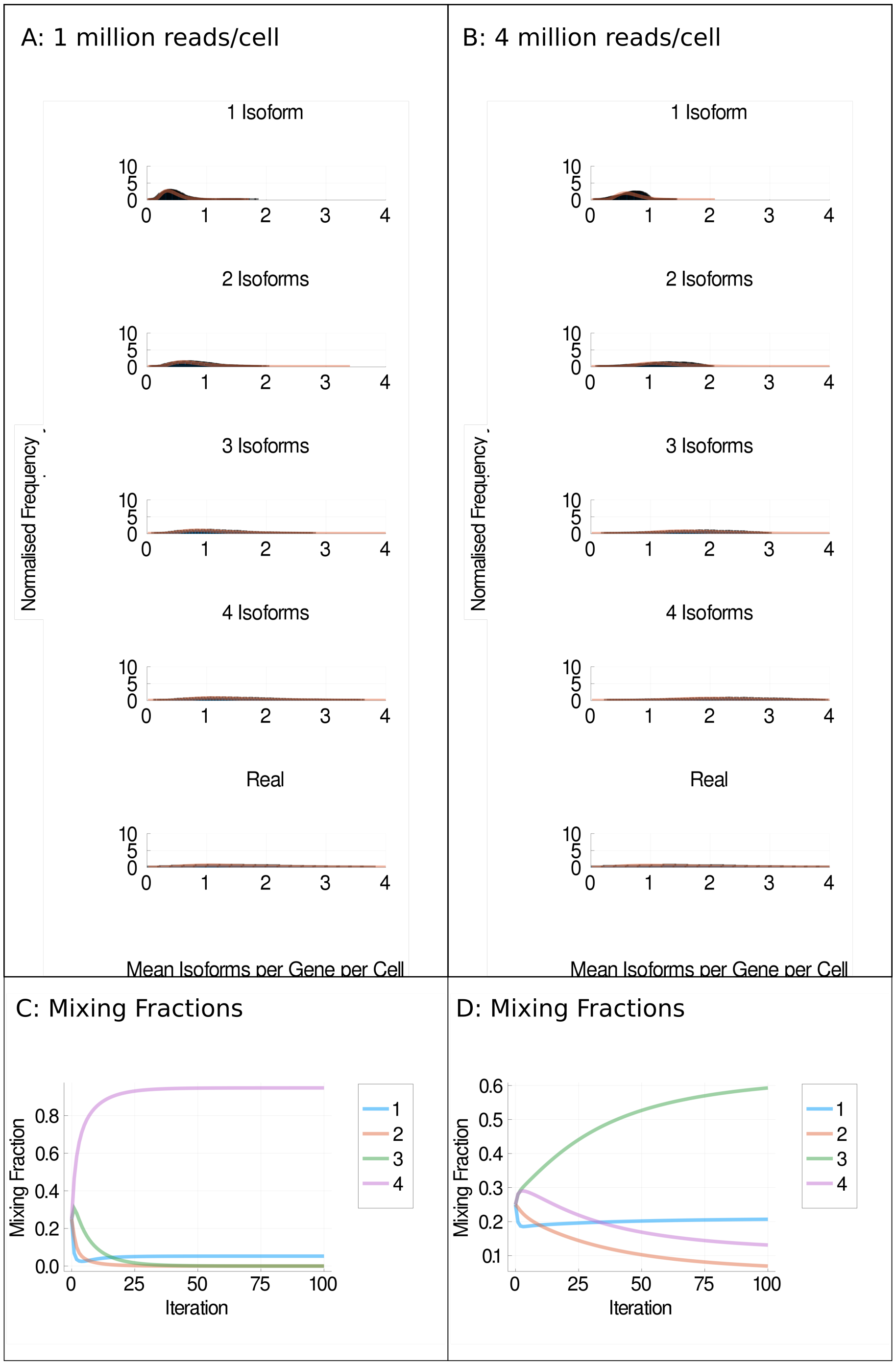

Fig. S26: Mixture models. **a** and **b** Distributions of detected isoforms per gene per cell (blue) and log normal fitted distributions (orange) for H1 cells sequenced at 1 million reads per cell (**a**) or 4 million reads per cell (**b**) under the inferred model [2]. **c** and **d** Mixing fractions vs iterations of expectation maximisation for 1 million reads per cell (**c**) and 4 million reads per cell (**d**). Each coloured line represents the distributions for one, two, three or four isoforms being simulated as expressed per gene per cell.

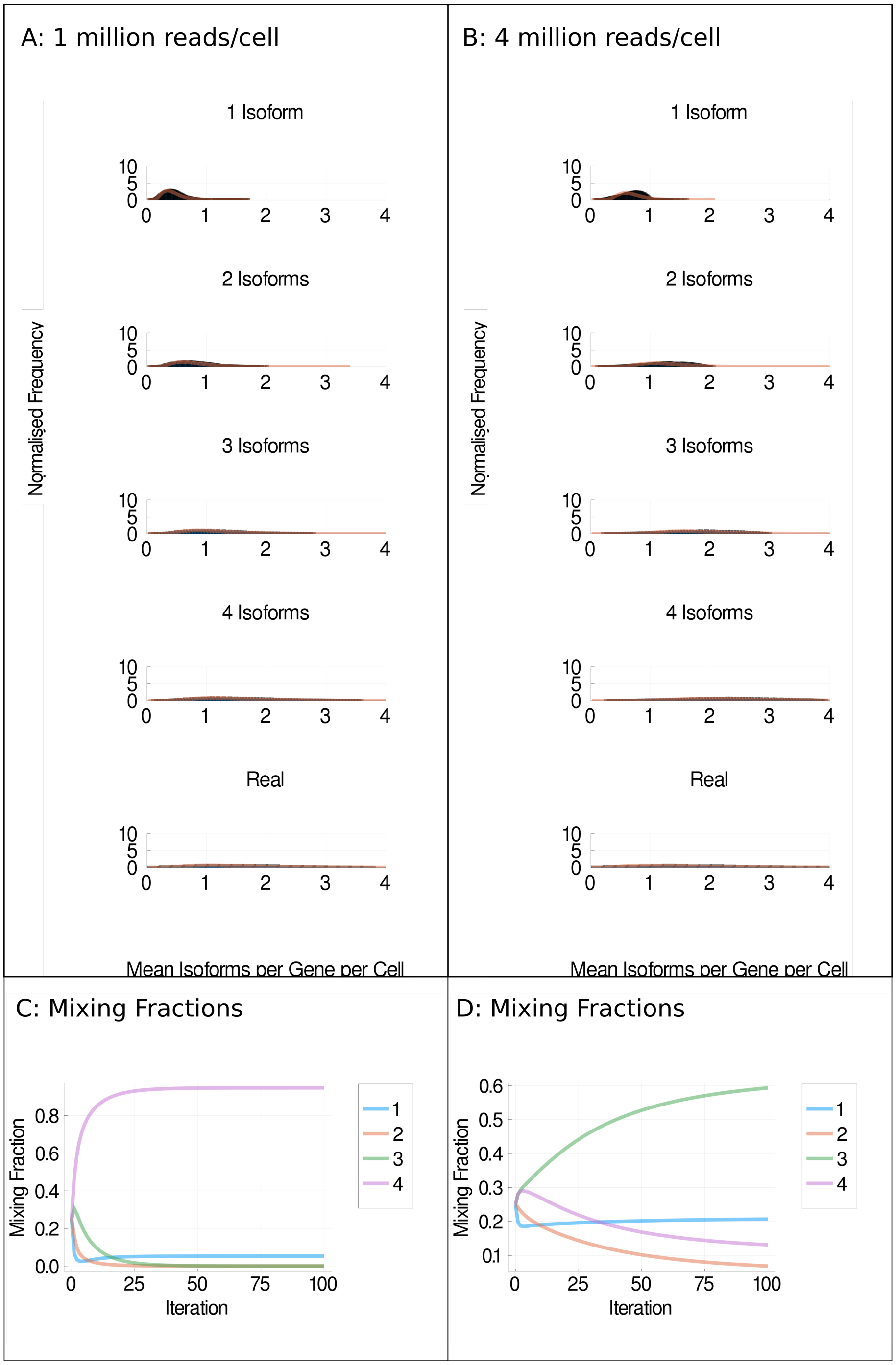

Fig. S27: Mixture models. **a** and **b** Distributions of detected isoforms per gene per cell (blue) and log normal fitted distributions (orange) for H1 cells sequenced at 1 million reads per cell (**a**) or 4 million reads per cell (**b**) under the cell variable model [2, 4]. **c** and **d** Mixing fractions vs iterations of expectation maximisation for 1 million reads per cell (**c**) and 4 million reads per cell (**d**). Each coloured line represents the distributions for one, two, three or four isoforms being simulated as expressed per gene per cell.

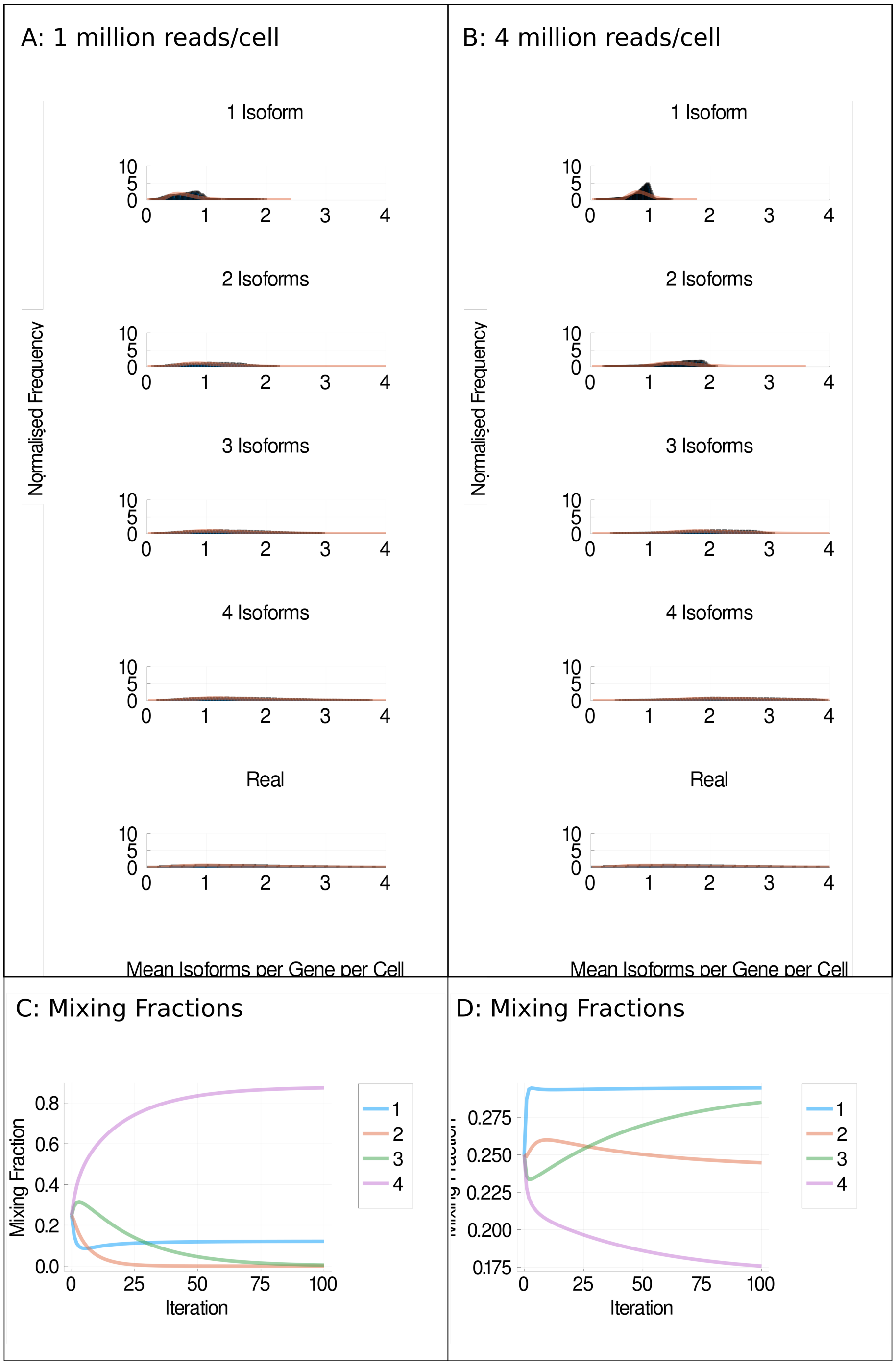

Fig. S28: Mixture models. **a** and **b** Distributions of detected isoforms per gene per cell (blue) and log normal fitted distributions (orange) for H9 cells sequenced at 1 million reads per cell (**a**) or 4 million reads per cell (**b**) under the Weibull model [2, 3]. **c** and **d** Mixing fractions vs iterations of expectation maximisation for 1 million reads per cell (**c**) and 4 million reads per cell (**d**). Each coloured line represents the distributions for one, two, three or four isoforms being simulated as expressed per gene per cell.

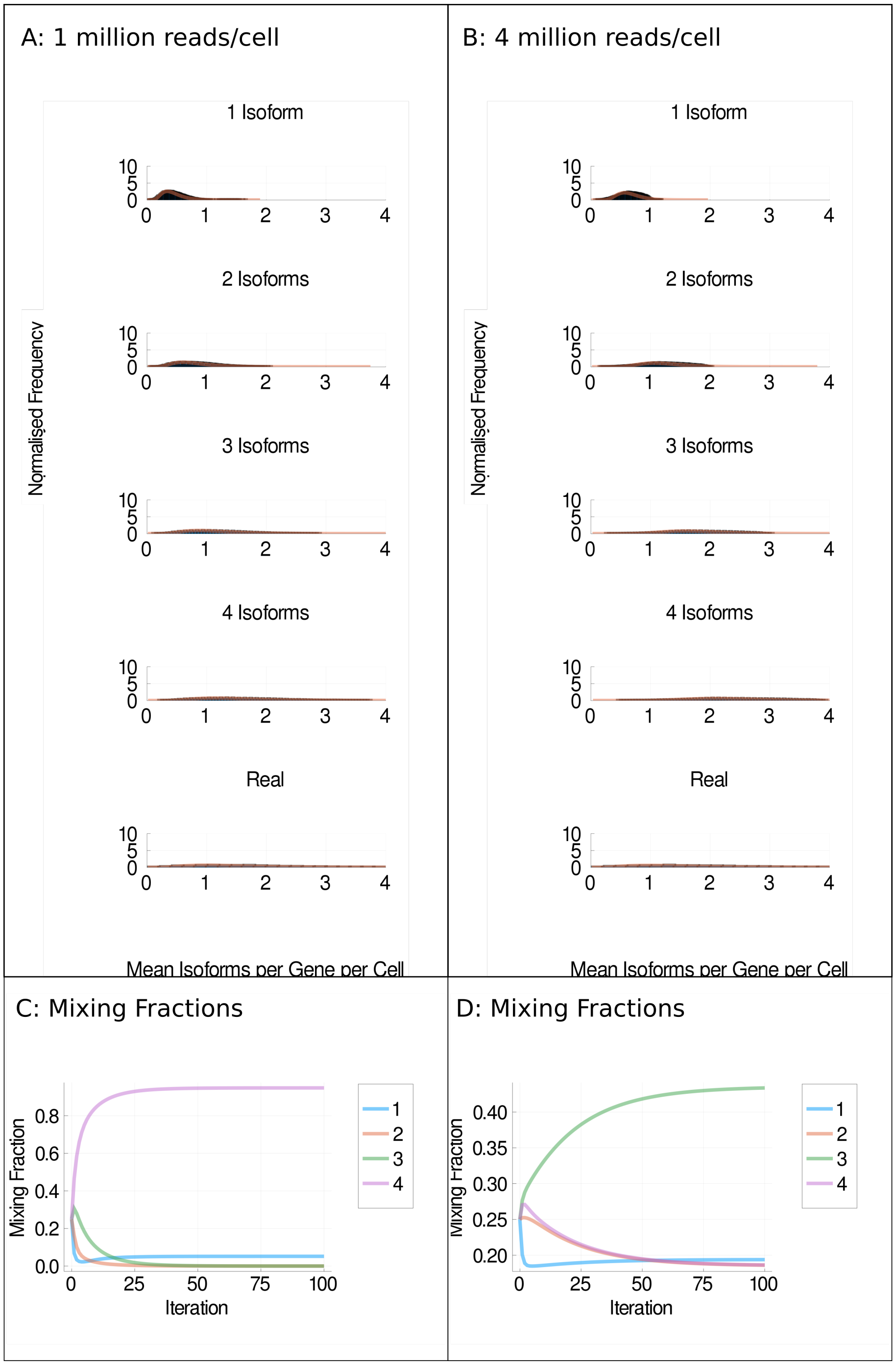

Fig. S29: Mixture models. **a** and **b** Distributions of detected isoforms per gene per cell (blue) and log normal fitted distributions (orange) for H9 cells sequenced at 1 million reads per cell (**a**) or 4 million reads per cell (**b**) under the random model [2]. **c** and **d** Mixing fractions vs iterations of expectation maximisation for 1 million reads per cell (**c**) and 4 million reads per cell (**d**). Each coloured line represents the distributions for one, two, three or four isoforms being simulated as expressed per gene per cell.

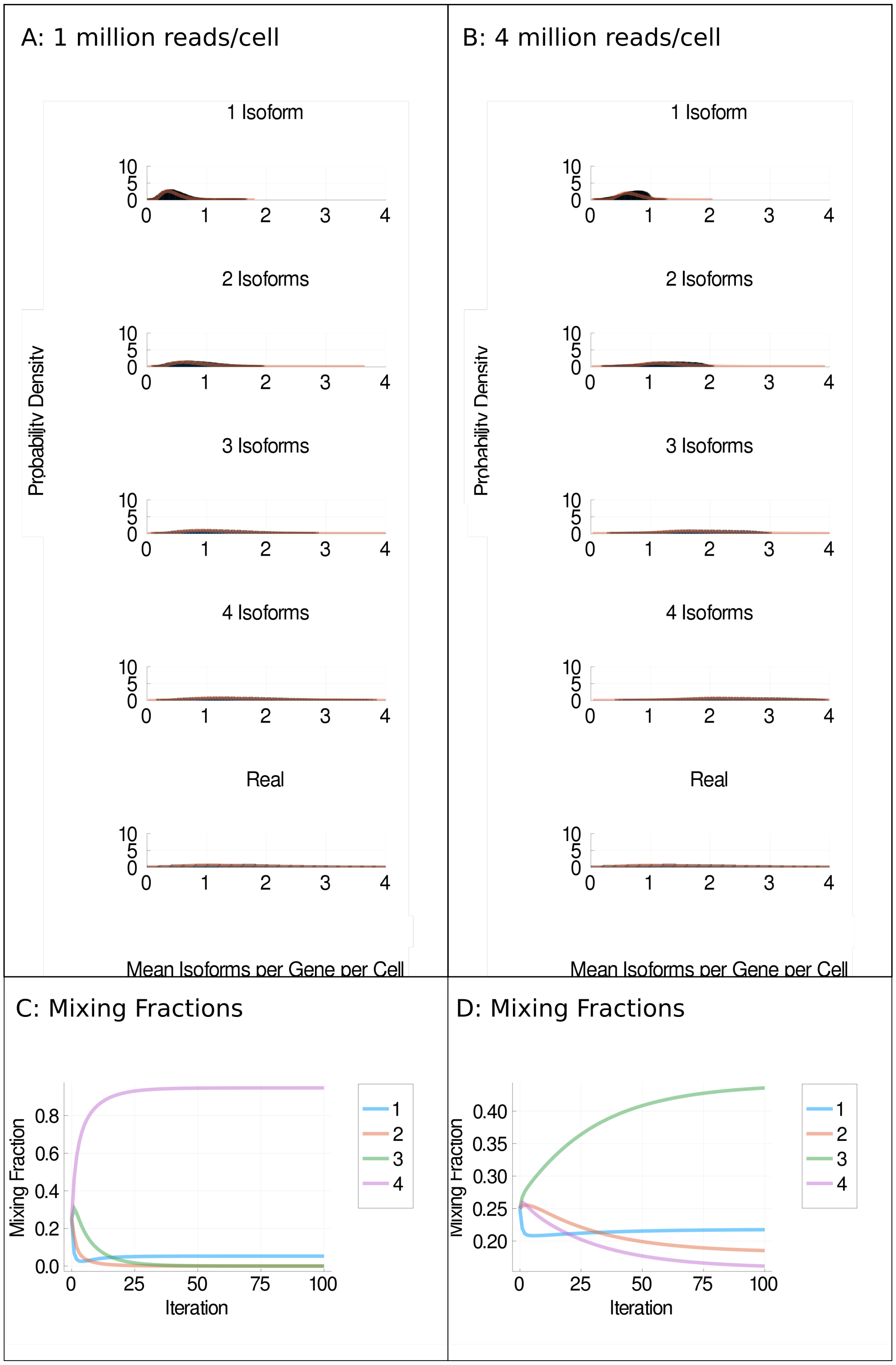

Fig. S30: Mixture models. **a** and **b** Distributions of detected isoforms per gene per cell (blue) and log normal fitted distributions (orange) for H9 cells sequenced at 1 million reads per cell (**a**) or 4 million reads per cell (**b**) under the inferred model [2]. **c** and **d** Mixing fractions vs iterations of expectation maximisation for 1 million reads per cell (**c**) and 4 million reads per cell (**d**). Each coloured line represents the distributions for one, two, three or four isoforms being simulated as expressed per gene per cell.

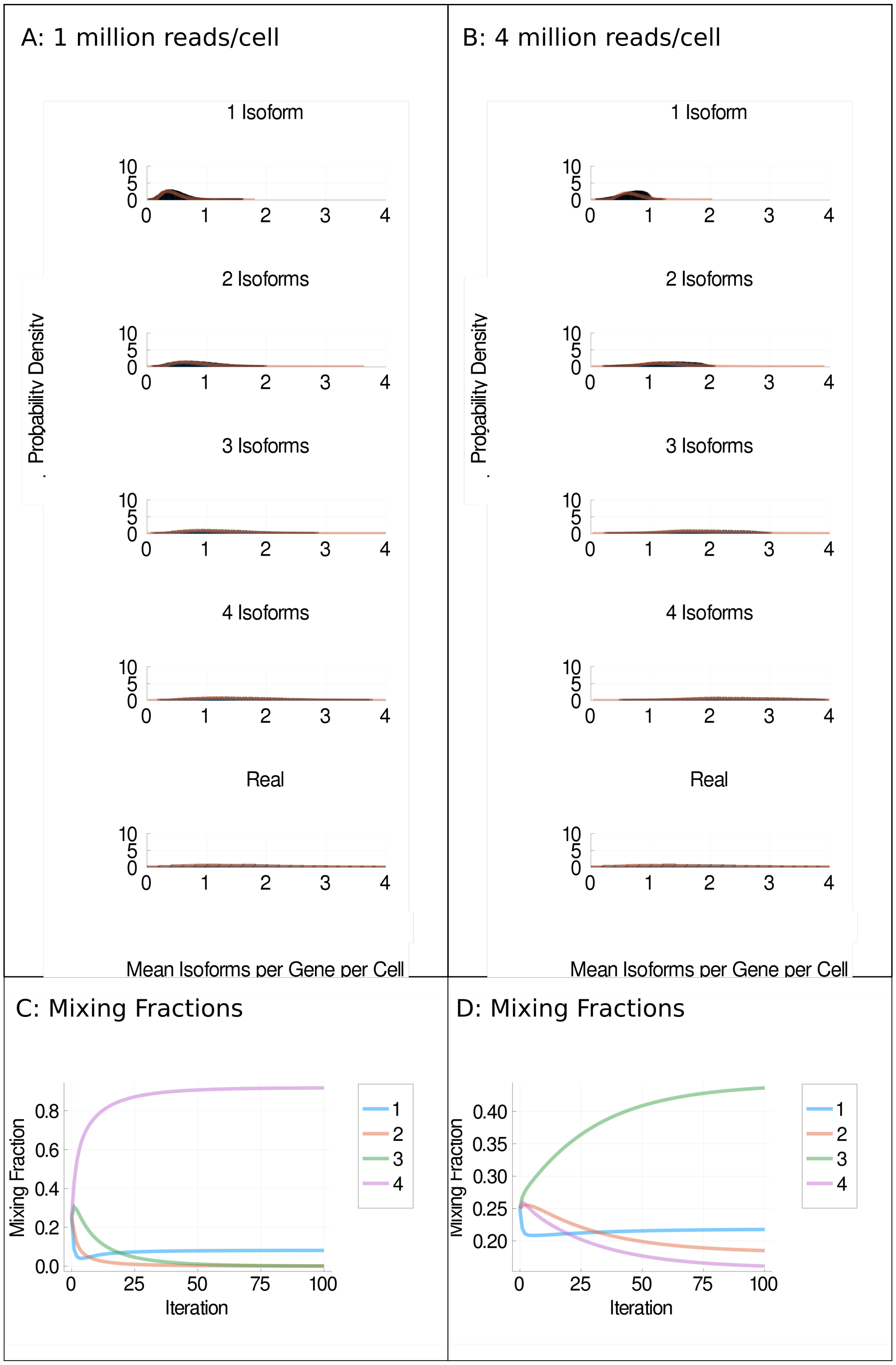

Fig. S31: Mixture models. **a** and **b** Distributions of detected isoforms per gene per cell (blue) and log normal fitted distributions (orange) for H9 cells sequenced at 1 million reads per cell (**a**) or 4 million reads per cell (**b**) under the cell variable model [2, 4]. **c** and **d** Mixing fractions vs iterations of expectation maximisation for 1 million reads per cell (**c**) and 4 million reads per cell (**d**). Each coloured line represents the distributions for one, two, three or four isoforms being simulated as expressed per gene per cell.

No. Isoforms Simulated	p-Value
1	0.0
2	0.0
3	0.0
4	0.999999

Table S1: Results of K-sample Anderson–Darling test, which tests whether multiple collections come from the same population. The test was applied to each row of graphs in Figure 5, in other words testing whether the distributions generated by different isoform choice models are significantly different.

No. Isoforms Simulated	p-Value
1	0.835737
2	0.997938
3	0.998721
4	0.99074

Table S2: Results of K-sample Anderson–Darling test, which tests whether multiple collections come from the same population. The test was applied to the simulation results generated using the Inferred Probabilities vs the Cell Variable models of isoform choice in Figure 5 to test whether the distributions generated by different isoform choice models significantly differ.

No. Isoforms Simulated	p-Value
1	0.0
2	0.0
3	0.0
4	1.0

Table S3: Results of K-sample Anderson–Darling test, which tests whether multiple collections come from the same population. The test was applied to each row of graphs in Supplementary Figure 8, in other words testing whether the distributions generated by different isoform choice models are significantly different.

No. Isoforms Simulated	p-Value
1	0.639939
2	0.959654
3	0.995236
4	0.999814

Table S4: Results of K-sample Anderson–Darling test, which tests whether multiple collections come from the same population. The test was applied to the simulation results generated using the Inferred Probabilities vs the Cell Variable models of isoform choice in Supplementary Figure 8 to test whether the distributions generated by different isoform choice models significantly differ.

No. Isoforms Simulated	p-Value
1	0.0
2	0.0
3	0.0
4	0.999999

Table S5: Results of K-sample Anderson–Darling test, which tests whether multiple collections come from the same population. The test was applied to each row of graphs in Supplementary Figure 10, in other words testing whether the distributions generated by different isoform choice models are significantly different.

No. Isoforms Simulated	p-Value
1	0.98348
2	0.95075
3	0.999405
4	0.995485

Table S6: Results of K-sample Anderson–Darling test, which tests whether multiple collections come from the same population. The test was applied to the simulation results generated using the Inferred Probabilities vs the Cell Variable models of isoform choice in Supplementary Figure 10 to test whether the distributions generated by different isoform choice models significantly differ.

No. Isoforms Simulated	p-Value
1	0.0
2	0.0
3	0.0
4	1.0

Table S7: Results of K-sample Anderson–Darling test, which tests whether multiple collections come from the same population. The test was applied to each row of graphs in Supplementary Figure 12, in other words testing whether the distributions generated by different isoform choice models are significantly different.

No. Isoforms Simulated	p-Value
1	0.932755
2	0.969666
3	0.999973
4	0.999753

Table S8: Results of K-sample Anderson–Darling test, which tests whether multiple collections come from the same population. The test was applied to the simulation results generated using the Inferred Probabilities vs the Cell Variable models of isoform choice in Supplementary Figure 12 to test whether the distributions generated by different isoform choice models significantly differ.

Data source	p-Value
H1 1 million reads	0.99808
H1 4 million reads	0.981612
H9 1 million reads	0.989299
H9 4 million reads	0.997866

Table S9: Results of K-sample Anderson–Darling test, which tests whether multiple collections come from the same population. The test was applied to the simulation results generated using the Normal, Bernoulli and p=0.25 models of isoform choice to test whether the distributions generated by different isoform choice models significantly differ.

References

[1] Kolodziejczyk, A.A., Kim, J.K., Tsang, J.C.H., Ilicic, T., Henriksson, J., Natarajan, K.N., Tuck, A.C., Gao, X., Bühler, M., Liu, P., Marioni, J.C., Teichmann, S.A.: Single cell RNA-sequencing of pluripotent states unlocks modular transcriptional variation. Cell Stem Cell **17**(4), 471–485 (2015). doi:10.1016/j.stem.2015.09.011. Accessed 2019-04-28

[2] Bacher, R., Chu, L.-F., Leng, N., Gasch, A.P., Thomson, J.A., Stewart, R.M., Newton, M., Kendziorski, C.: SCnorm: robust normalization of single-cell RNA-seq data. Nature Methods **14**(6), 584–586 (2017). doi:10.1038/nmeth.4263. Accessed 2017-04-17

[3] Hu, J., Boritz, E., Wylie, W., Douek, D.C.: Stochastic principles governing alternative splicing of RNA. PLoS Computational Biology **13**(9), 1005761 (2017). doi:10.1371/journal.pcbi.1005761. Accessed 2018-11-23

[4] Velten, L., Anders, S., Pekowska, A., Järvelin, A.I., Huber, W., Pelechano, V., Steinmetz, L.M.: Single-cell polyadenylation site mapping reveals 3' isoform choice variability. Molecular Systems Biology **11**(6), 812 (2015). doi:10.15252/msb.20156198. Accessed 2019-04-28
