# Supplementary material for: Obstacles to detecting isoforms using full-length scRNA-seq data
Source: Genome Biol. 2020 Mar 23;21:74. doi: 10.1186/s13059-020-01981-w (PMC7087381; doi:10.1186/s13059-020-01981-w)
Supplement: Supplementary file 2 — Additional file 2 Review history. [file 13059_2020_1981_MOESM2_ESM.docx]

**First round**

**Reviewer 1**

In this manuscript, Westoby and colleagues set out to investigate whether alternative splicing analysis with scRNA-seq is feasible with current methods and technologies, and what are the current hurdles. This is certainly a topic of interest to the community, and I think that this paper raises some important points; however, there are also parts where I feel that the claims are a bit too broad, or where the methods need better explanation. Below are more detailed comments.

1. The title of the paper is, in my opinion, quite a bit broader than the topics that are actually discussed in detail. For example:

* The choice of scRNA-seq protocol likely has a large impact on the isoform detection ability, and to my understanding the paper assumes that the data was collected withb a full-length scRNA-seq protocol. This is reasonable if one is interested in alternative splicing, but the additional challenges and/or opportunities posed by the popular 3' UMI-based protocols or other/newer full-length protocols are not mentioned.

* The evaluations in the paper only focus on whether the right number of isoforms are detected - not whether they are the correct ones, or whether the estimated abundances are accurate. These are also important aspects of studying alternative splicing. Perhaps some form of overlap measure could be used to compare the sets of true and observed isoforms, rather than just counting them?

* The main results illlustrate that since not all expressed isoforms are likely to be captured and assigned reads in the sequencing/quantification process, it is likely that the number of expressed isoforms in a single cell is underestimated. However, would it still be possible to detect alternative splicing, e.g., if in each of two cells one detects a single isoform, but the detected isoforms are clearly distinct?

* Alternative splicing could be studied in other ways than by quantifying full transcripts, e.g., by considering individual splicing events. Would this type of analysis be equally affected?

2. I think that it would be helpful to have a figure providing a schematic of the simulation procedure. In particular, it was not clear to me how the gene counts in individual cells (in the original count matrix) are used. If I understand correctly, the selection of isoforms in a single cell depends only on the probabilities estimated from the overall isoform expression, and also the dropout estimation uses only the overall expression levels. If a certain isoform is selected to be expressed in a given cell, but the observed count is zero, is it then considered a false negative, and if so, wouldn't this assume that there is no underlying heterogeneity of the isoform expression across cells in a sample (so that all zeros are technical dropouts)?

3. While the dropout rate depends on the overall expression of the isoform, the probability of a false negative (pFN) is considered to be constant. Could you elaborate on the rationale behind this (do you assume that the quantification software will misassign either all or none of the reads from a transcript)?

4. The authors observe that the model of isoform choice affects the performance. However, I am wondering if the effect is mainly related to the model itself, or the resulting expression levels of the retained isoforms (i.e., some models retain more likely highly expressed isoforms). In other words, if one would keep the model constant, but increase the expression of the selected isoforms, would that affect the performance to a similar extent?

5. The formula for P(Choice) on page 13 does not follow from equation (2) unless P(Choice)=P(not Choice)

6. In the dropout probability estimation based on the Michaelis-Menten model, what are the "expression" values? Raw counts, normalized counts, log-transformed values?

**Reviewer 2**

In this paper, authors address several issues involving single-cell level isoform studies, providing further mathematical insight and proof for some of them, and highlighting their importance to guide interpretation of single-cell isoform study results. Given that isoform analysis at single-cells is currently one of the less explored single-cell RNAseq applications, the present study is both timely and highly relevant. The study complements previous efforts in pointing out limitations of these studies and raising some very interesting questions regarding the feasibility of a fully reliable single-cell isoform-level study. I would therefore like to congratulate authors for their efforts.

In order to improve the manuscript, some issues should be addressed:

Results section:

P5, L12: "Third, we simulate dropouts based on a Michaelis-Menten model described by Andrews and Hemberg [9]. Fourth, we simulate quantification errors based on isoform detection error estimates based on work by Westoby et al [8]."

In the present study, authors successfully create a model for single-cell isoform-level counts. However, the manuscript does not include any negative control model, i.e. repeating simulations without dropout and quantification error modelling. Including such a model would be a good way to assess the correctness of the model, that is, to what extent the results observed can be attributed to these two phenomena (dropouts and quantification inaccuracy), or they are generated by some of the assumptions in the author's model. Ultimately, this could help clarify some of the ambiguities in the results, such as in P10, L43.

P6, L49: "As our interest in this study is the detected number of isoforms per gene per cell, we are only interested in quantification errors which lead to changes in the number of isoforms detected."

Although authors successfully model and measure the impact of true positive and true negative events, accuracy of expression estimates and how this changes the ranking of isoforms in each cell or cell group are also worth evaluation. This is particularly relevant considering the statement, recurrent throughout the paper, that splicing typically occurs by the expression of a "main" or dominant isoform, and the lower expression of other, alternative variants. Would inaccurate quantification change these rankings, and how often? Would this impact which of the isoforms is identified as the dominant one? An interesting addition would be for authors to complete the quantification accuracy study by modelling frequencies of ranking changes, i.e. in each cell as well as mean ranking changes, together with the frequency of main isoform identification changes (where the "main" is the most highly expressed isoform in a cell, i.e. ranking 1). This would also add additional perspective on the isoform choice model sections.

P7, L21: "Due to the high number of dropouts in scRNA-seq, many expressed isoforms do not generate reads and thus would be called as unexpressed by a 100% accurate isoform quantification tool, leading to an under-estimate of the number of isoforms present."

Authors raise a very interesting point when discussing false negative events and their relationship to dropouts. Indeed, quantification software accuracy is not the only factor driving false negatives, as software tools can only quantify what is present in the sequencing library. A previous study addressing the impact of coverage bias in 3' vs full-length libraries in isoform detection, done via simulation of reads (Arzalluz-Luque and Conesa, 2018) did not model read-level dropouts. However, dropouts are unlikely to occur due to consistently missing reads from all available copies of a full-length transcripts, and are more probably generated via failure to capture sufficient isoform-unique reads to enable identification of that isoform via subsequent mapping and quantification. This would be an interesting addition for the present study, namely modelling different proportions of missing reads from a bulk-RNAseq ground truth, assuming no dropouts in this condition. This would help further sustain this statement, and would be a valuable addition to the study by answering the question of what is the limit of missing isoform-unique reads required to ulimately cause an isoform-level dropout.

P7, L49: "It is possible that different mechanisms of isoform choice at the cellular level could alter our ability to correctly detect what isoforms are present in scRNA-seq. Because there is uncertainty over the mechanism of isoform choice within single cells, we implement four different models of isoform choice in our simulations."

Isoform choice is performed at the cell level by biological criteria that are not entirely known, as authors accurately state in their study. However, it is very likely that isoform choice variability in single-cell RNAseq studies is directly influenced by read mapping properties, i.e. spliced read abundances, junction coverages… Which are fundamental aspects of computational isoform detection when using short read technologies. Probabilistically choosing isoforms to be expressed is an interesting way to demonstrate that researchers do not possess the knowledge required to understand the underlying mechanisms driving splicing. However, doing so without primarily discussing read generation biases and mapping fails to capture the complexity of isoform detection, since author's simulation strategy assumes expression estimates of genes with at least four isoforms to be a ground truth. Further insight could probably be obtained by completing the quantification error section via a read-level dropout simulation as indicated above, however, some of these issues should be discussed in this section of the manuscript, as well as the discussion (at P11, L18).

Discussion section:

P12, L11: "Long read technologies could in theory enable 100% accurate isoform quantification, if issues due to a high base calling error rate could be overcome."

The main limitation of long reads in single-cell is that the technology suffers from a lack of depth per cell, not only sequencing error biases, as simulated and discussed in a previous study (Arzalluz-Luque and Conesa 2018). These results should be incorporated into the discussion section to further clarify that detection of multiple isoforms per gene per cell is practically unfeasible at the moment using long-read technologies, and is the main force hindering long-read single-cell studies. This same limitation currently makes it unfeasible to perform a quantitative study using long reads at the single-cell level.

P11, L50: "Unfortunately, extremely deeply sequenced datasets (eg. >10 million reads per cell) are likely to suffer more with PCR artefacts and potentially a higher false positive rate of isoform detection."

The recently published Smart-seq3 preprint would be a relevant addition to further expand this section, indicating how the new technology could improve isoform studies in single-cell RNAseq (Hagemann-Jensen et al. 2019, bioRxiv) by incorporating 5' UMIs into a full-length short-read protocol.

More generally, although this was stated at the beginning of the manuscript, it would be interesting to add to the discussion that an accurate definition of the transcriptome, namely by generating tissue or system specific transcriptomes and/or by long-read definition and curation of isoforms to eliminate false positives and artifacts, would greatly aid isoform quantification. To some extent, this would certainly help reduce the effect of dropouts, quantification inaccuracy, etc. in single-cell RNAseq, as well as gain further biological insight of cell-level isoform expression.

Figures:

Figure 1B: it would be useful to have the same number of bins and y axis scales on both histograms, helping authors make their point that dropout probabilities are lower with higher sequencing depth and easing comparison of the histograms and beta distributions.

**Response to reviewers**

In this manuscript, Westoby and colleagues set out to investigate whether alternative splicing analysis with scRNA-seq is feasible with current methods and technologies, and what are the current hurdles. This is certainly a topic of interest to the community, and I think that this paper raises some important points; however, there are also parts where I feel that the claims are a bit too broad, or where the methods need better explanation. Below are more detailed comments.

We thank the reviewer for their constructive criticisms and positive assessment. We address each of their comments inline below:

1. The title of the paper is, in my opinion, quite a bit broader than the topics that are actually discussed in detail. For example:

* The choice of scRNA-seq protocol likely has a large impact on the isoform detection ability, and to my understanding the paper assumes that the data was collected with a full-length scRNA-seq protocol. This is reasonable if one is interested in alternative splicing, but the additional challenges and/or opportunities posed by the popular 3' UMI-based protocols or other/newer full-length protocols are not mentioned.

We agree with the reviewer that the manuscript would be improved by making it more explicit that we exclusively use full-length scRNA-seq data and by discussing the challenges and opportunities posed by 3’ UMI-based protocols. To address this, we have updated the title of the manuscript to ‘Obstacles to Detecting Isoforms Using Full-Length scRNA-seq Data’ and have clarified in the introduction (page 2, line 4) that we use full-length scRNA-seq data in our simulations. In addition, we have added a paragraph in our Discussion (page 14, line 16 onwards) in which we explain our reasons for using full-length scRNA-seq data and discuss the challenges associated with using UMI-based protocols for isoform detection. In this paragraph, we also mention the new full-length protocol SMART-seq3, and consider how SMART-seq3 could be beneficial to the problem of isoform detection.

* The evaluations in the paper only focus on whether the right number of isoforms are detected - not whether they are the correct ones, or whether the estimated abundances are accurate. These are also important aspects of studying alternative splicing. Perhaps some form of overlap measure could be used to compare the sets of true and observed isoforms, rather than just counting them?

We agree that the correctness of the isoforms detected is highly relevant when studying alternative splicing using scRNA-seq. We are grateful for the reviewer’s suggestion that an overlap measure would be informative in this context, and have devised an overlap measure based on the proportion of isoforms detected in our simulations that are present in the ground truth (page 3, line 30 onwards). We have updated Figures 2 & 3 so that they include overlap measure plots and discuss the implications of the overlap metrics in the manuscript text (page 3, line 41 and page 4, line 36). Additionally, Supplementary Figures 4, 5, 6, 8, 10, 12 and 21 now display overlap metrics.

The reviewer also raises the importance of the accuracy of estimated abundances when studying alternative splicing using scRNA-seq. We agree that the accuracy of estimated abundances is important, however our simulations do not directly model the abundances of isoforms and only report whether or not isoforms are detected. Adding the ability to realistically model isoform abundance to our simulations would be challenging, especially given that there is no ground truth dataset for how highly expressed isoforms typically are in individual cells to base our model on. As we do not consider isoform abundance in our study, we have changed the manuscript title to: ‘Obstacles to Detecting Isoforms Using Full-Length scRNA-seq Data’ to make the scope of our manuscript more explicit. We have also added a paragraph to our Discussion in which we explain our rationale for exclusively considering isoform detection in our study (see page 14, line 43).

* The main results illlustrate that since not all expressed isoforms are likely to be captured and assigned reads in the sequencing/quantification process, it is likely that the number of expressed isoforms in a single cell is underestimated. However, would it still be possible to detect alternative splicing, e.g., if in each of two cells one detects a single isoform, but the detected isoforms are clearly distinct?

This is an excellent question. It is indeed sometimes possible to detect alternative splicing if in each of two cells one detects a single isoform, and the detected isoforms are clearly distinct. An example of a gene for which this is likely to be possible is illustrated below:

*Figure 1: Kpnb1 isoforms. Image taken from*

*http://www.ensembl.org/Mus_musculus/Gene/Summary?db=core;g=ENSMUSG00000001440;r*

*=11:97159714-97187881.*

Let’s imagine that reads from the 3’ end of Kpnb1-201 are detected in one cell in an scRNA-seq experiment, and reads from the retained intron at the 5’ end of Kpnb1-203 are detected in another cell. In this situation, we can say with pretty high confidence that alternative splicing is occurring across this population of cells.

However, Kpnb1 is an unusual example, because Kpnb1 has highly dissimilar isoforms. Consequently we would expect it to be relatively easy to determine which isoform generated the detected reads. A more typical example is Hdlbp:

*Figure 2: Hdlbp isoforms. Image taken from*

*http://www.ensembl.org/Mus_musculus/Gene/Summary?db=core;g=ENSMUSG00000034088;r*

*=1:93405940-93478815.*

Although Hdlbp has some clearly distinct isoforms, some of the isoforms are highly similar. Consequently, we might expect it to be challenging to determine which isoform(s) produced the reads detected in individual cells. For example, let’s imagine one cell produces Hdlbp-201 and one cell produces Hdlbp-204. If there are no reads from the 3’ end of the transcripts, it might not be possible to say with high confidence whether or not alternative splicing is occurring, because we can’t say with high confidence which isoform(s) are being produced in each cell.

Our view is that it is sometimes possible to detect alternative splicing using scRNA-seq, but we would expect to generally underestimate the number of splicing events due to confounding caused by dropouts. In addition, we would expect some confounding due to isoform detection errors (such as the Hdlbp example above), although our simulations suggest this may be a more minor confounder. We have added a sentence in the Discussion to clarify this point (see page 11, line 25).

Alternative splicing could be studied in other ways than by quantifying full transcripts, e.g., by considering individual splicing events. Would this type of analysis be equally affected?

As the reviewer notes, isoform quantification is not the only way to study alternative splicing. We focussed on isoform quantification as opposed to exon centric approaches (eg. MISO (Katz et al. 2010) ) because an independent benchmark has been carried out for isoform quantification tools using scRNA-seq (Westoby et al. 2018) , whereas to the best of our knowledge such a benchmark does not exist for exon centric approaches. We suspect that exon centric approaches would also be confounded by the high rate of dropouts in scRNA-seq and have added a paragraph on this topic in the Discussion (see page 14, line 31). It is less clear whether exon centric approaches would be affected by software errors when calling splicing events and different isoform choice models in the same way as isoform quantification tools.

2. I think that it would be helpful to have a figure providing a schematic of the simulation

procedure.

We thank the reviewer for their suggestion. Figure 1 is now a figure providing a schematic of the simulation procedure, mentioned in the text at page 3, line 15.

In particular, it was not clear to me how the gene counts in individual cells (in the original count matrix) are used. If I understand correctly, the selection of isoforms in a single cell depends only on the probabilities estimated from the overall isoform expression, and also the dropout estimation uses only the overall expression levels. If a certain isoform is selected to be expressed in a given cell, but the observed count is zero, is it then considered a false negative, and if so, wouldn't this assume that there is no underlying heterogeneity of the isoform expression across cells in a sample (so that all zeros are technical dropouts)?

The reviewer is correct in their understanding of the simulation process. The reviewer correctly raises the issue that in our simulations, we assume that there are no biological dropouts, with the exception of isoforms for which zero expression is recorded in all or nearly all cells, which we call as unexpressed (see page 16, line 21, Methods step 1). Therefore, in our simulations we assume that all dropouts are technical. This assumption is unlikely to always be correct and is a respect in which our simulations are overly simplistic. However, we note that at present, to the best of our knowledge there is no reliable methodology that would enable us to distinguish between biological and technical dropouts. Indeed, distinguishing between biological and technical dropouts is essentially the goal of imputation approaches, however a recent benchmark of imputation tools found that such approaches often generate a high rate of false positive results (Andrews and Hemberg 2018) . Distinguishing between technical and biological dropouts is an interesting and important problem which we believe merits further research, however we feel that solving this challenging problem goes beyond the scope of our current manuscript. Instead, we simulate situations in which there are no biological dropouts and explain in the Discussion that our current inability to accurately model biological dropouts is a limitation of our approach (see page 12, line 3 onwards).

3. While the dropout rate depends on the overall expression of the isoform, the probability of a false negative (pFN) is considered to be constant. Could you elaborate on the rationale behind this (do you assume that the quantification software will misassign either all or none of the reads from a transcript)?

As the reviewer correctly identifies, we assume that the probability of false negative and false positive events are constant values in our simulations. This is almost certainly an over-simplification. The probability of a particular isoform experiencing a false positive or false negative event is probably influenced by many factors, including but not limited to gene length, percentage of sequence shared with other isoforms from same parent gene, magnitude of expression and GC content. Unfortunately, less research has been done on modelling isoform detection errors compared to modelling technical dropouts. Thus, attempts to incorporate factors such as magnitude of expression and GC content into our model of isoform detection errors would probably rely on inaccurate estimates and best guesses of the effect of these factors on isoform quantification software performance. Rather than take such an approach, we have chosen to assume that the probabilities of false positive and false negative events are constant, which we recognise is probably an over-simplification. We discuss this on page 12, line 35, and explain that further research is required to improve simulations of quantification errors.

4. The authors observe that the model of isoform choice affects the performance. However, I am wondering if the effect is mainly related to the model itself, or the resulting expression levels of the retained isoforms (i.e., some models retain more likely highly expressed isoforms). In other words, if one would keep the model constant, but increase the expression of the selected isoforms, would that affect the performance to a similar extent?

The reviewer raises the interesting question of why different models of isoform choice alter our ability to detect isoforms in our simulations. We agree with the reviewer that it seems likely that some models’ preferences for picking more highly expressed isoforms might be responsible for this phenomenon. In our simulations, we do not directly model isoform abundance, however we do model probability of dropout. As it is known that the probability of dropout is inversely correlated with magnitude of expression (Kharchenko et al. 2014) , we use probability of dropout as an indicator of magnitude of expression here.

We first ask whether it is indeed true that our different models of isoform choice have different preferences for picking highly expressed isoforms. Highly expressed isoforms would be expected to have a lower probability of dropout, so we investigate this question by asking what the probability of dropout typically is when one, two, three or four isoforms are picked using the Weibull, Random, Inferred Probability and Cell Variability models. Our results are shown below:

*Figure 3: Distributions of the probabilities of dropout for the isoforms chosen using the Weibull model when one, two, three or four isoforms are chosen from the H1 hESCs sequenced at 4 million reads per cell.*

*Figure 4: Distributions of the probabilities of dropout for the isoforms chosen using the Random model when one, two, three or four isoforms are chosen from the H1 hESCs sequenced at 4 million reads per cell.*

*Figure 5: Distributions of the probabilities of dropout for the isoforms chosen using the Inferred Probability model when one, two, three or four isoforms are chosen from the H1 hESCs sequenced at 4 million reads per cell.*

*Figure 6: Distributions of the probabilities of dropout for the isoforms chosen using the Cell Variability model when one, two, three or four isoforms are chosen from the H1 hESCs sequenced at 4 million reads per cell.*

Clearly, when we ask our models to pick one isoform, some models preferentially pick isoforms with a much lower probability of dropout (and hence a higher magnitude of expression) compared to others. Of the four models considered here, when the models are asked to choose just one isoform, the Weibull model most often picks isoforms with a probability of dropout close to zero. We suspect this is why the Weibull model detects the most isoforms and generates the most accurate overlap statistics when one isoform is picked per gene per cell. Based on these results, we agree with the reviewer that the most likely reason that our models differ in performance is due to their differing preferences for picking highly expressed isoforms. The reviewer asks whether, when the model is kept constant and the expression of isoforms is increased, the performance is affected to a similar extent. To address this question, we return to our approach illustrated in Figures 3b, c and d in the manuscript. In Figures 3b, c, and d, the probabilities of dropout are sampled from various Beta distributions. Due to the inverse relationship between the probability of dropout and magnitude of expression, this could be regarded as equivalent to sampling isoforms from various magnitude of expression distributions.

In Figure 8 below, we can see that under all four models of isoform choice, as the Beta distribution sampled from becomes more skewed towards zero, the ability to accurately detect the correct number of isoforms (in this case, one isoform) improves. This further supports the reviewer’s hypothesis that the magnitude of expression of the picked isoforms is the main reason for the differences in the performance of different models of isoform choice.

*Figure 7: The Beta distributions used to sample dropout probabilities from.*

*Figure 8: Distributions of the mean number of isoforms detected per gene per cell under different isoform choice models when dropout probabilities are sampled from the Beta distributions in Figure 7.*

We have added these figures to our Supplementary Figures (see Supplementary Figures 15-18), and discussed these results in our manuscript at page 8, line 4 onwards, and in our Discussion at page 13, line 8..

5. The formula for P(Choice) on page 13 does not follow from equation (2) unless P(Choice)=P(not Choice)

We thank the reviewer for spotting the error in our formula, which we have now corrected. During the review process, we also recognised an error in our implementation of the Weibull model, which we have also corrected.

6. In the dropout probability estimation based on the Michaelis-Menten model, what are the "expression" values? Raw counts, normalized counts, log-transformed values?

We use counts per million (CPM), and have clarified this in the Methods (see page 18, line 46 onwards).

**Reviewer 2**

In this paper, authors address several issues involving single-cell level isoform studies, providing further mathematical insight and proof for some of them, and highlighting their importance to guide interpretation of single-cell isoform study results. Given that isoform analysis at single-cells is currently one of the less explored single-cell RNAseq applications, the present study is both timely and highly relevant. The study complements previous efforts in pointing out limitations of these studies and raising some very interesting questions regarding the feasibility of a fully reliable single-cell isoform-level study. I would therefore like to congratulate authors for their efforts.

We are grateful to the reviewer for their constructive criticisms and their positive assessment of our manuscript. We address each of their concerns inline below:

In order to improve the manuscript, some issues should be addressed:

Results section:

P5, L12: "Third, we simulate dropouts based on a Michaelis-Menten model described by Andrews and Hemberg [9]. Fourth, we simulate quantification errors based on isoform detection error estimates based on work by Westoby et al [8]."

In the present study, authors successfully create a model for single-cell isoform-level counts. However, the manuscript does not include any negative control model, i.e. repeating simulations without dropout and quantification error modelling. Including such a model would be a good way to assess the correctness of the model, that is, to what extent the results observed can be attributed to these two phenomena (dropouts and quantification inaccuracy), or they are generated by some of the assumptions in the author's model. Ultimately, this could help clarify some of the ambiguities in the results, such as in P10, L43.

We thank the reviewer for their suggestion that negative control models should be included in our manuscript. We have updated our supplementary figures so that Supplementary Figures 1-3 show our simulation results for H1 hESCs when 1) neither dropouts nor quantification errors are simulated, 2) no dropouts are simulated and 3) no quantification errors are simulated. We refer to our new supplementary figures at page 3, line 16 in the manuscript.

P6, L49: "As our interest in this study is the detected number of isoforms per gene per cell, we are only interested in quantification errors which lead to changes in the number of isoforms detected."

Although authors successfully model and measure the impact of true positive and true negative events, accuracy of expression estimates and how this changes the ranking of isoforms in each cell or cell group are also worth evaluation. This is particularly relevant considering the statement, recurrent throughout the paper, that splicing typically occurs by the expression of a "main" or dominant isoform, and the lower expression of other, alternative variants. Would inaccurate quantification change these rankings, and how often? Would this impact which of the isoforms is identified as the dominant one? An interesting addition would be for authors to complete the quantification accuracy study by modelling frequencies of ranking changes, i.e. in each cell as well as mean ranking changes, together with the frequency of main isoform identification changes (where the "main" is the most highly expressed isoform in a cell, i.e. ranking 1). This would also add additional perspective on the isoform choice model sections.

We agree with the reviewer that assessing the impact of inaccurate isoform quantification on isoform ranking would be an interesting study. Our existing simulation framework only simulates whether isoforms are expressed and whether they are detected in a binary fashion. In other words, isoform abundance is not directly simulated. Extending our simulation framework to simulate abundances would not be trivial, as we would need to model isoform abundance in individual cells, model PCR amplification bias and model the impact of abundance on the probability of isoform quantification errors. Most challenging would be developing a model to simulate abundances in individual cells, as there is no ground truth dataset available to base this model on. As we do not consider isoform abundance in our study, we have changed the manuscript title to: ‘Obstacles to Detecting Isoforms Using Full-Length scRNA-seq Data’ to make the scope of our manuscript more explicit. We have also added a paragraph to our Discussion in which we explain our rationale for exclusively considering isoform detection in our study (see page 14, line 43).

P7, L21: "Due to the high number of dropouts in scRNA-seq, many expressed isoforms do not generate reads and thus would be called as unexpressed by a 100% accurate isoform quantification tool, leading to an under-estimate of the number of isoforms present." Authors raise a very interesting point when discussing false negative events and their relationship to dropouts. Indeed, quantification software accuracy is not the only factor driving false negatives, as software tools can only quantify what is present in the sequencing library. Our previous study addressing the impact of coverage bias in 3' vs full-length libraries in isoform detection, done via simulation of reads (Arzalluz-Luque and Conesa, 2018) did not model read-level dropouts. However, dropouts are unlikely to occur due to consistently missing reads from all available copies of a full-length transcripts, and are more probably generated via failure to capture sufficient isoform-unique reads to enable identification of that isoform via subsequent mapping and quantification. This would be an interesting addition for the present study, namely modelling different proportions of missing reads from a bulk-RNAseq ground truth, assuming no dropouts in this condition. This would help further sustain this statement, and would be a valuable addition to the study by answering the question of what is the limit of missing isoform-unique reads required to ulimately cause an isoform-level dropout.

We agree with the reviewer that it would be interesting to investigate the impact of downsampling bulk RNA-seq data on our ability to detect isoforms. In the context of our present study, an interesting question is: If bulk RNA-seq data is downsampled to the same depth as matched scRNA-seq data, are we more or less able to detect isoforms present in the bulk RNA-seq data using the matched scRNA-seq data or the downsampled bulk RNA-seq data? If we are equally able to detect isoforms in the downsampled and the scRNA-seq dataset, this suggests that our reduced ability to detect isoforms in scRNA-seq data can be entirely explained by a reduction in sequencing depth relative to bulk RNA-seq. This would imply that our reduced ability to detect isoforms was entirely due to consistently missing reads from all available copies of a full-length transcripts, as the reviewer suggests, and/or because quantification tools are less able to resolve between similar isoforms when there are less reads available to quantify. In contrast, if there is a difference in our ability to detect isoforms in the downsampled and scRNA-seq datasets, it suggests that there is some fundamental difference between downsampled bulk RNA-seq and scRNA-seq data. The difference could be biological (ie. there could be heterogeneous isoform expression in individual cells) or technical (for example, the enzymatic reactions associated with library preparation may have reduced efficiency when there is a lower amount of starting material). There could additionally be interplay between biological and/or technical factors and isoform quantification software performance. For example, if isoform expression is more uniform in one data type than the other, we might expect isoform quantification software to be able to quantify the reads in that data type more accurately. To investigate this question, we required dataset of matched bulk and scRNA-seq datasets. We selected a dataset of mouse Embryonic Stem Cells (mESCs) for which the bulk RNA-seq data was sequenced to approximately 44 million reads and the scRNA-seq data was sequenced to approximately 7 million reads (Kolodziejczyk et al. 2015) . We then randomly downsampled reads from the bulk RNA-seq dataset 50 times to 7 million reads. The outcome was 50 downsampled fastq files containing 7 million random reads, comparable to 50 of the cells sequenced in the matched scRNA-seq dataset.

We considered the isoforms detected in the original bulk RNA-seq dataset to be the ground truth and calculated the mean overlap fraction with the ground truth for each gene (see Methods in main manuscript, line [insert] for details). These results are displayed below:

*Figure 9: Boxplots of the mean overlap for each gene in the downsampled bulk and matched scRNA-seq datasets. The mean overlaps for each gene are overlaid on the boxplots as black points.*

Interestingly, we found that the mean overlap fraction was significantly higher (p<2.2 x 10^-16, Welch two sample t-test) for the downsampled bulk RNA-seq than for the matched scRNA-seq. Therefore, we are more able to detect isoforms present in the bulk RNA-seq using the downsampled bulk data relative to the matched scRNA-seq data. This result suggests that the reduction in our ability to detect isoforms in scRNA-seq relative to bulk RNA-seq cannot entirely be explained by a lower number of reads. We hypothesise that the reduction in performance over the downsampled datasets is likely due to a combination of heterogeneous isoform expression between cells, and technical differences between bulk and scRNA-seq.

We have added Figure 9 above to our Supplementary Figures (see Supplementary Figure 7) and discuss this figure in our Results section, at page 6, line 7 onwards, and in Methods at page 20, line 1.

P7, L49: "It is possible that different mechanisms of isoform choice at the cellular level could alter our ability to correctly detect what isoforms are present in scRNA-seq. Because there is uncertainty over the mechanism of isoform choice within single cells, we implement four different models of isoform choice in our simulations."

Isoform choice is performed at the cell level by biological criteria that are not entirely known, as authors accurately state in their study. However, it is very likely that isoform choice variability in single-cell RNAseq studies is directly influenced by read mapping properties, i.e. spliced read abundances, junction coverages… Which are fundamental aspects of computational isoform detection when using short read technologies. Probabilistically choosing isoforms to be expressed is an interesting way to demonstrate that researchers do not possess the knowledge required to understand the underlying mechanisms driving splicing. However, doing so without primarily discussing read generation biases and mapping fails to capture the complexity of isoform detection, since author's simulation strategy assumes expression estimates of genes with at least four isoforms to be a ground truth. Further insight could probably be obtained by completing the quantification error section via a read-level dropout simulation as indicated above, however, some of these issues should be discussed in this section of the manuscript, as well as the discussion (at P11, L18).

We thank the reviewer for raising the interesting point that there is likely to be some interplay between isoform choice and quantification errors, which we do not capture in our simulations. We discuss this issue in our Discussion (see page 13, line 22 onwards), and raise the possibility that the gap in isoform detection ability discovered in the read-level dropout simulation could be partly due to this interplay.

Discussion section:

P12, L11: "Long read technologies could in theory enable 100% accurate isoform quantification, if issues due to a high base calling error rate could be overcome."

The main limitation of long reads in single-cell isI that the technology suffers from a lack of depth per cell, not only sequencing error biases, as simulated and discussed in our previous work (Arzalluz-Luque and Conesa 2018). These results should be incorporated into the discussion section to further clarify that detection of multiple isoforms per gene per cell is practically unfeasible at the moment using long-read technologies, and is the main force hindering long-read single-cell studies. This same limitation currently makes it unfeasible to perform a quantitative study using long reads at the single-cell level.

We thank the reviewer for bringing this to our attention, and have edited the Discussion text at page 12, line 24 to raise this point.

P11, L50: "Unfortunately, extremely deeply sequenced datasets (eg. >10 million reads per cell) are likely to suffer more with PCR artefacts and potentially a higher false positive rate of isoform detection."

The recently published Smart-seq3 preprint would be a relevant addition to further expand this section, indicating how the new technology could improve isoform studies in single-cell RNAseq (Hagemann-Jensen et al. 2019, bioRxiv) by incorporating 5' UMIs into a full-length short-read protocol.

We have updated our Discussion to include a paragraph in which we discuss the newly developed SMART-seq3 protocol and the impact it might have on isoform detection (see page 14, line 23).

More generally, although this was stated at the beginning of the manuscript, it would be interesting to add to the discussion that an accurate definition of the transcriptome, namely by generating tissue or system specific transcriptomes and/or by long-read definition and curation of isoforms to eliminate false positives and artifacts, would greatly aid isoform quantification. To some extent, this would certainly help reduce the effect of dropouts, quantification inaccuracy, etc. in single-cell RNAseq, as well as gain further biological insight of cell-level isoform expression.

We agree with the reviewer that a more accurate definition of the transcriptome is key to enabling accurate isoform detection and quantification. We have added a few sentences incorporating the reviewer’s points in the Discussion at page 12, line 30.

Figures:

Figure 1B: it would be useful to have the same number of bins and y axis scales on both histograms, helping authors make their point that dropout probabilities are lower with higher sequencing depth and easing comparison of the histograms and beta distributions.

We thank the reviewer for their suggestion and have updated the histograms so that the same number of bins and y axis scales are used in both histograms (now in Figure 2a of the manuscript).

Bibliography

Andrews, T.S. and Hemberg, M. 2018. False signals induced by single-cell imputation. [version 2; peer review: 4 approved]. *F1000Research* 7, p. 1740.

Katz, Y., Wang, E.T., Airoldi, E.M. and Burge, C.B. 2010. Analysis and design of RNA sequencing experiments for identifying isoform regulation. *Nature Methods* 7(12), pp. 1009–1015.

Kharchenko, P.V., Silberstein, L. and Scadden, D.T. 2014. Bayesian approach to single-cell differential expression analysis. *Nature Methods* 11(7), pp. 740–742.

Kolodziejczyk, A.A., Kim, J.K., Tsang, J.C.H., et al. 2015. Single Cell RNA-Sequencing of Pluripotent States Unlocks Modular Transcriptional Variation. *Cell Stem Cell* 17(4), pp. 471–485.

Westoby, J., Herrera, M.S., Ferguson-Smith, A.C. and Hemberg, M. 2018. Simulation-based benchmarking of isoform quantification in single-cell RNA-seq. *Genome Biology* 19(1), p. 191.

**Second Round**

**Reviewer 1**

The authors have addressed my concerns in a careful and satisfactory manner. I have no further comments.

**Reviewer 2**

Authors have addressed my concerns and did an excellent job in improving the manuscript.
